# Synthesis of 3-alkenylindoles through regioselective C–H alkenylation of indoles by a ruthenium nanocatalyst

**DOI:** 10.3762/bjoc.16.16

**Published:** 2020-01-29

**Authors:** Abhijit Paul, Debnath Chatterjee, Srirupa Banerjee, Somnath Yadav

**Affiliations:** 1 Department of Chemistry, Indian Institute of Technology (ISM), Dhanbad, 826004, Jharkhand, India; 2 Department of Chemistry, Bethune College, Bidhan Sarani, Kolkata, 700006, West Bengal, India

**Keywords:** alkenylation, C–H activation, heterogeneous catalysis, nanocatalysis, ruthenium catalysis

## Abstract

3-Alkenylindoles are biologically and medicinally very important compounds, and their syntheses have received considerable attention. Herein, we report the synthesis of 3-alkenylindoles via a regioselective alkenylation of indoles, catalysed by a ruthenium nanocatalyst (RuNC). The reaction tolerates several electron-withdrawing and electron-donating groups on the indole moiety. Additionally, a “robustness screen” has also been employed to demonstrate the tolerance of several functional groups relevant to medicinal chemistry. With respect to the Ru nanocatalyst, it has been demonstrated that it is recoverable and recyclable up to four cycles. Also, the catalyst acts through a heterogeneous mechanism, which has been proven by various techniques, such as ICPMS and three-phase tests. The nature of the Ru nanocatalyst surface has also been thoroughly examined by various techniques, and it has been found that the oxides on the surface are responsible for the high catalytic efficiency of the Ru nanocatalyst.

## Introduction

**Figure 1:**
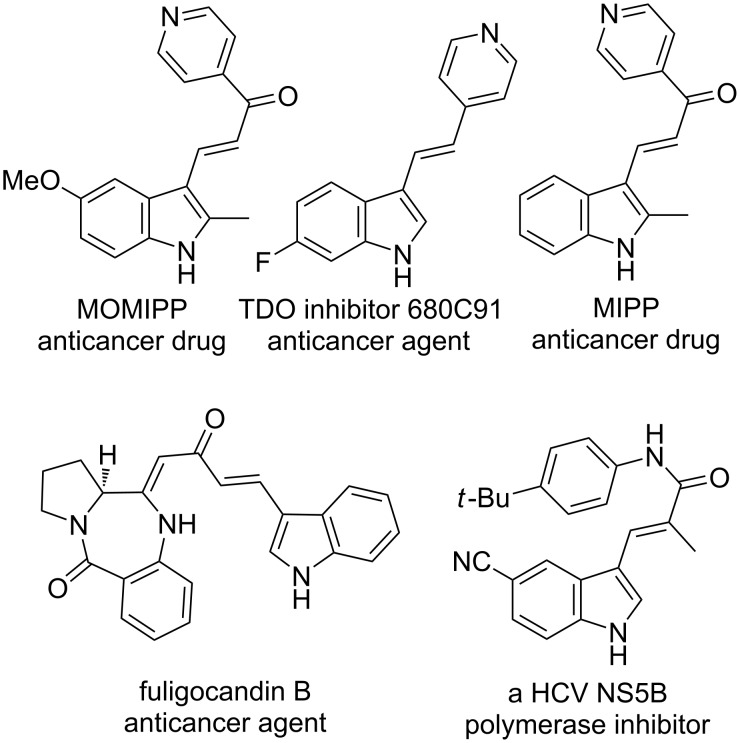
Biologically and medicinally important 3-alkenylindoles.

The synthesis of functionalised indole ring systems has received significant attention over the years, as these are the vital structural motifs of several biologically and medicinally important compounds [1–4]. Also, 3-alkenylindoles act as fundamental building blocks for the synthesis of materials such as carbazoles [5–6], indole alkaloids [7–9], etc. Again, 3-alkenylindoles also form the core of proposed anticancer compounds like MIPP and MOMIPP [10], fuligocandin B [2], the TDO inhibitor 680C91 [11], and a HCV NS5B polymerase inhibitor, which has been proposed as a drug against hepatitis C (Figure 1) [12].

**Scheme 1:**
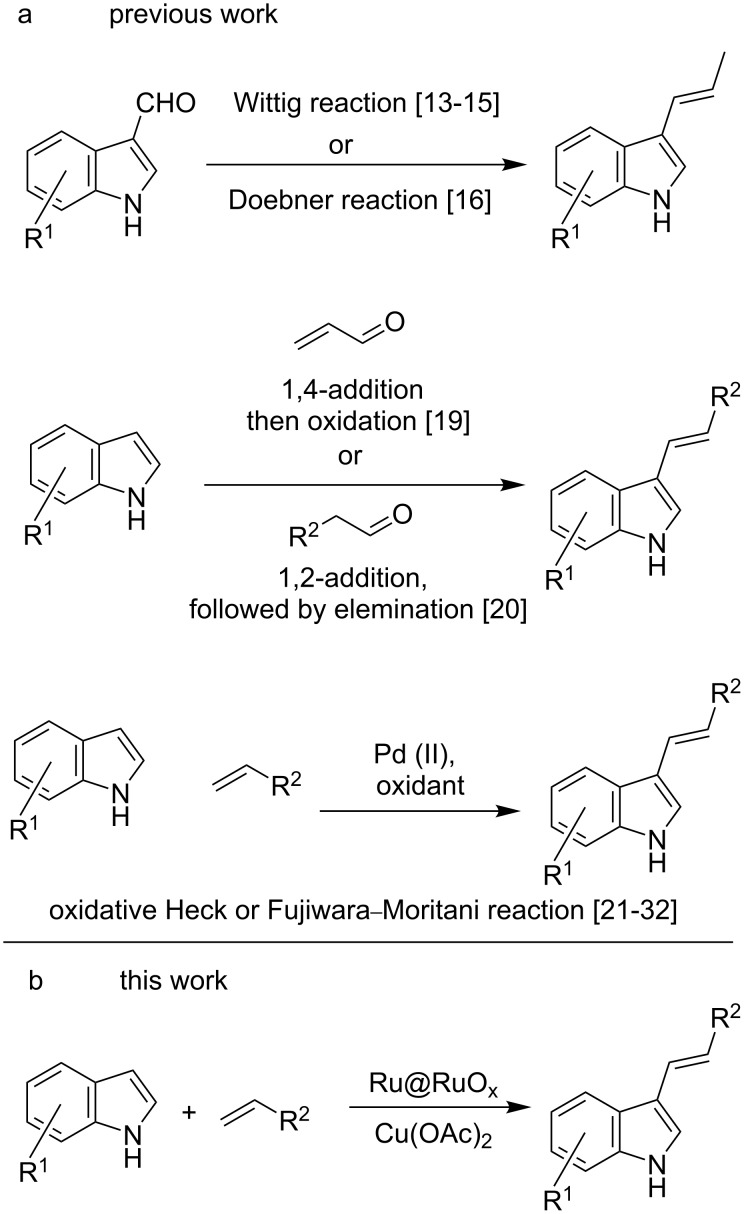
a) Previous and b) present work related to the synthesis of 3-alkenylindoles.

The syntheses of 3-alkenylindoles can generally be classified into the following three categories: (i) by Wittig or Doebner reaction of indoles bearing a 3-aldehyde group; (ii) by 1,4- or 1,2-addition of α,β-enones or carbonyl compounds, followed by oxidation or elimination, respectively; (iii) by Pd-catalysed oxidative coupling of indoles with activated alkenes. Several groups have used Wittig reactions for the synthesis of 3-alkenylindoles [13–15]. Another variant that uses the Doebner condensation was reported by Singh and co-worker, who condensed indole-3-carbaldehyde with phenylacetic acid in the presence of pyridine as the solvent/base and piperidine as the catalyst [16]. However, this strategy was associated with several shortcomings, as it required two to four successive steps for the synthesis of the 3-indolecarbaldehydes starting form indoles, low yields, a narrow scope, and selectivity issues among the geometrical isomers, which led to troubles in purification [17–18]. As an example for the second category, Jiao and co-workers developed an organocatalytic C3–H alkenylation of indoles by the reaction of indoles with α,β-unsaturated aldehydes in presence of morpholin-4-ium trifluoroacetate as a catalyst and a stoichiometric amount of DDQ to achieve oxidative dehydrogenation [19]. Recently, Maji and co-workers reported the synthesis of 3-alkenylindoles from indoles and α-hydrogen-containing alkyl-/arylaldehydes by successive Brønsted acid/base catalysis (Scheme 1) [20].

The third category, which is also the most explored and popular one, involves the Pd-catalysed Fujiwara–Moritani or oxidative dehydrogenative Heck reaction via dual C–H activation [21–24]. One of the early examples of this reaction, reported by Gaunt and co-workers, involved the regioselective, solvent-controlled C3 alkenylation of indoles with alkenes containing electron-withdrawing groups, using Pd(OAc)_2_ as catalyst and Cu(OAc)_2_ as oxidant [25]. Since then, several variants of the reaction involving Pd catalysis and various oxidants have been reported for the synthesis of 3-alkenylindoles. For example, Chen et al. and Huang et al. independently reported the C3 alkenylation of indoles using Pd(OAc)_2_ and Pd(II)/polyoxometallate, respectively, as a catalyst and molecular oxygen as the oxidant [26–27]. Verma and co-workers used the reaction between indoles and alkenes in the presence of a Pd(OAc)_2_ catalyst, a Cu(OAc)_2_ oxidant, and a 2-(1-benzotriazolyl)pyridine ligand [28]. Noël and co-workers reported the C3–H olefination of indoles using Pd(OAc)_2_ as a catalyst and molecular oxygen as the oxidant under continuous flow conditions [29]. Jia et al. reported the synthesis of 3-alkenylindoles using Pd(OAc)_2_ as the catalyst and MnO_2_ as the oxidant under ball milling conditions [30]. Das and co-workers reported the C3–H alkenylation of 7-azaindole using Pd(OAc)_2_ as a catalyst, Ph_3_P as a ligand, and Cu(OTf)_2_ as an oxidative cocatalyst, with molecular oxygen as the oxidant [31]. Carrow and co-workers reported mechanistic, kinetic, and selectivity studies of the C–H alkenylation of indole with *n*-butylacrylate in the presence of thioether ligands [32].

In the context of C–H activation reactions, the catalyst of choice has mostly been Pd [33–34]. However, as part of the search for newer and more cost-efficient catalysts, other transition metals, such as Ru, have also been explored, with some favourable results [35–40]. Other very important aspects of Ru catalysts are mechanistic aspects, which has also favoured their exploration for directing group-assisted C–H activation reactions [40]. With respect to non-directed Ru nanoparticle-catalysed reactions, there are few reports. For example, the supported Ru-catalysed regiospecific C(sp^2^)–H arylation of benzo[*h*]quinolines and the addition of vinylsilanes to the C–H bonds of α-tetralones were reported by Inoue and co-workers [41–42]. Pieters et al. reported the Ru nanoparticle-catalysed C–H deuteration reaction of aza compounds [43–44]. Again, a Ru nanoparticle-catalysed C–H selenylation of indoles was reported by Lin et al. [45]. Herein, we report the Ru-catalysed regioselective synthesis of C3 alkenylindoles using a near-naked, surfactant-free, and recyclable Ru nanocatalyst in a heterogeneous manner.

## Results and Discussion

### Synthesis and characterisation of the Ru nanocatalyst

The surfactant- and stabiliser-free RuNC was synthesised photochemically, based on a procedure that we have previously reported for the synthesis of Pd nanoparticles [46–47]. The synthesised RuNC, obtained directly after photolysis, was characterised by TEM (Figure S1, Supporting Information File 1), which showed polydispersed spherical particles of a size distribution mainly in the range of 10–25 nm, with a mean diameter of 15 nm. The size distribution of the particles from Figure S1, Supporting Information File 1, is presented in Figure S3, Supporting Information File 1. TEM–EDX confirmed the nanoparticles to be those of Ru (Figure S2, Supporting Information File 1). Further, the Ru nanoparticles were separated by centrifugation and characterised in more detail. The TEM analysis of the isolated Ru nanoparticles showed considerable agglomeration of the individual nanoparticles (Figure S4, Supporting Information File 1). The HRTEM–SAED diffraction image showed the presence of several crystalline phases, including those for Ru(0) and RuO_2_ (Figure S5, Supporting Information File 1). More specifically, the crystalline planes (101), (210), (103), and (200), corresponding to the interlayer spacings of 2.10, 1.38, 1.24, and 1.18 Å, respectively, could be identified for Ru(0), and the crystalline planes (200) and (221), corresponding to the interlayer spacings of 2.38 and 1.60 Å for RuO_2_, could be identified. The TEM–EDX analysis (Figure S6, Supporting Information File 1) distinctly showed the presence of Ru. The experiment also showed the presence of a small amount of oxygen, which could be attributed to the presence of some surface oxides.

Powder X-ray diffraction (Figure S7, Supporting Information File 1) of the isolated nanoparticles showed several amorphous phases, along with diffraction peaks for Ru(0) at 2Θ = 38.3, 43.4, 57.7, 69.0, 77.8, and 84.8°, which could be designated to the (100), (101), (102), (210), (103), and (201) planes, respectively (JCPDS file no. 00-006-0663). The isolated RuNC was also analysed by XPS, which showed peaks at 280.0 and 284.7 eV (Figure S8, Supporting Information File 1), corresponded to the 3d_5/2_ and 3d_3/2_ peak regions of ruthenium (Figure S9, Supporting Information File 1). This could be deconvoluted to the peaks for Ru(0) at 279.8 and 283.8 eV and RuO_2_ at 280.5 and 284.6 in the sample (Figure S10, Supporting Information File 1) [48–49]. Additionally, in the XPS experiment, the peaks corresponding to O 1s at 529.7 (Figure S8, Supporting Information File 1) could also be detected which, unequivocally pointed at the presence of RuO_x_ in addition to the Ru(0) species. The ruthenium:oxygen ratio was found to be 3:1 from the XPS elemental ratio (Figure S8, Supporting Information File 1). Further confirmation of the presence of surface oxides was obtained through IR spectroscopy, which showed a Ru–O stretching peak at 462 cm^−1^ (Figure S9, Supporting Information File 1). IR spectroscopy also revealed that the surface of the catalyst contained a negligible amount of organic compounds and was therefore appropriately clean. Thus, the RuNC that we used in this study can be characterised as Ru@RuO_x_ where the bulk of the Ru nanocatalyst is zerovalent state and contained ruthenium oxides/hydroxides on the surface.

### Ru nanocatalyst-catalysed C–H alkenylation of indoles

After the synthesis of the RuNC, we explored the catalytic activity of the material in C–H alkenylation reactions of indole (**1a**). Initial optimisation of the conditions for the alkenylation reactions were carried out employing indole (**1a**), methyl acrylate (**2a**), and 3 mg of the RuNC. Different oxidants as well as solvents were explored for the reaction. From the optimisation reactions and the control experiments, it was concluded that the reaction with Cu(OAc)_2_ as the oxidant in DMF/DMSO, 9:1, v/v at 130 °C for 12 h were the best conditions, affording the product **3a** in 81% yield (Table 1, entry 4) after 12 h. Control reactions using RuCl_3_ or the absence of any catalyst in the presence of Cu(OAc)_2_ were also carried out, which demonstrated that the RuNC was essential for the reaction. Another control reaction was also carried out using [Ru(*p*-cymene)Cl_2_]_2_ as a homogeneous catalyst, but this also did not lead to the formation of the desired product.

**Table 1:** Control experiments and optimisation of the conditions for the alkenylation of indole (**1a**).

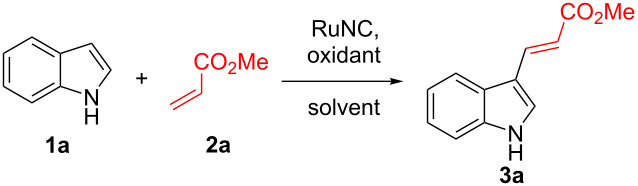				

entry	oxidant	solvent	time (h)	yield of **3a** (%)^a^

1^b^	Cu(OAc)_2_	dioxane	24	32
2^b^	Cu(OAc)_2_	DMF	12	63
3^b^	Cu(OAc)_2_	DMSO	12	32
4^b^	Cu(OAc)_2_	DMF/DMSO^c^	12	81
5^b^	–	DMF/DMSO^c^	24	–
6^b^	K_2_S_2_O_8_	DMF/DMSO^c^	24	27
7^b^	K_3_Fe(CN)_6_	DMF/DMSO^c^	24	–
8^d^	Cu(OAc)_2_	DMF/DMSO^c^	24	–
9^e^	Cu(OAc)_2_	DMF/DMSO^c^	12	17
10^f^	Cu(OAc)_2_	DMF/DMSO^c^	12	–

^a^Isolated yield. ^b^Reaction conditions: **1** (1 mmol), **2** (2 mmol), oxidant (1.8 mmol), solvent (5 mL), RuNC (3 mg), Ar, 12–24 h, 130 °C. ^c^Ratio = 9:1. ^d^No catalyst was added. ^e^RuCl_3_⋅3H_2_O (0.2 mol %) was used as a catalyst. ^f^[Ru(*p*-cymene)Cl_2_]_2_ (0.2 mol %) was used as a catalyst.

**Scheme 2:**
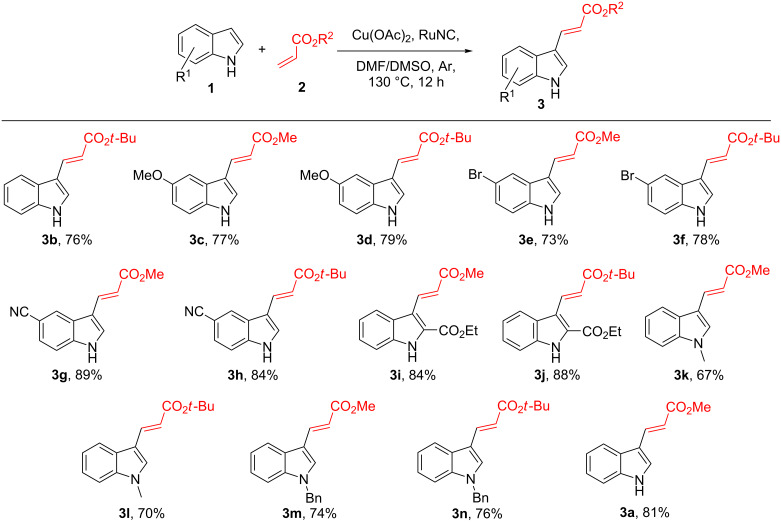
Substrate scope for the C–H alkenylation of the indoles **1**. Reaction conditions: **1** (1 mmol), **2** (2 mmol), oxidant (1.8 mmol), DMF/DMSO, 9:1, v/v (5 mL), RuNC (3 mg), Ar, 130 °C, 12 h. All yields are isolated yields.

After establishing the optimum conditions for the reaction, we carried out the alkenylation of several indole derivatives **1** with different acrylates **2** under the standard conditions using the RuNC. The results are summarised in Scheme 2. The reactions led to the successful regioselective C3 alkenylation of different indoles **1** with substrates **2** bearing both electron-donating and electron-withdrawing groups on the indole moiety. The reaction was also successful with a bromo-substituted substrates **3e** and **3f**, demonstrating that the methodology was suitable for substrates with the potential for further late-stage modification. Steric effects were also explored with C2-substituted substrate **3i** and **3j**, and no significant decline in product formation was observed.

To further test the functional group tolerance of the reaction, we employed a modified version of the “robustness screen” method promulgated by Glorius and co-workers [50–52]. For this purpose, the reaction of indole (**1a**) was carried out with **2b** in the presence of several additives bearing different functional groups (Table 2). It was found that the reactions tolerated carboxylic acid, ketone, halogen (Cl, Br, I, and F), aldehyde, amide, primary amine, secondary amine, and phenolic functional groups to a reasonably acceptable extent.

**Table 2:** Robustness screen of the synthesis of 3-alkenylindole **3b**.^a^

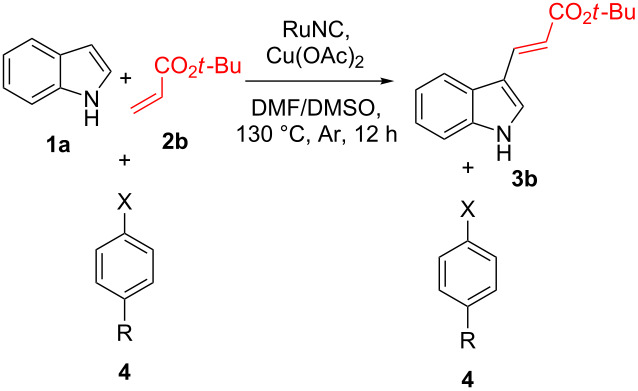			

Entry	Additive	**3b** (%)^b^	**4** (%)^c^

1	**4a**, R = H, X = COOH	74	94
2	**4b**, R = Cl, X = COCH_3_	73	89
3	**4c**, R = Cl, X = CHO	71	79
4	**4d**, R = H, X = NHCOCH_3_	74	84
5	**4e**, R = H, X = NH_2_	71	90
6	**4f**, R = H, X = NHPh	70	89
7	**4h**, R = OCH_3_, X = OH	68	75
8	**4i**, R = H, X = I	67	60
9	**4j**, R = CH_3_, X = Br	71	83
10	**4k**, R = NH_2_, X = F	63	88

^a^The reactions were performed under standard conditions in the presence of 1 mmol of **4**. ^b^Isolated yields. ^c^Recovered material.

### Recovery and recyclability of the Ru nanocatalyst

The reusability and recyclability of the solid RuNC was then tested in the reaction of **1a** with **2a**. The catalyst was recovered from the C–H alkenylation reaction and reused in subsequent reactions, with up to eight cycles (Figure S11, Supporting Information File 1). To recover the catalyst, the reaction mixture was diluted with ethyl acetate, and then water was added to it, which resulted in the dissolution of the soluble copper salts. Then, the mixture was centrifuged at 17000 rpm, and the supernatant liquid was decanted. The residue was successively washed thrice more with water, and finally the centrifuge tube was dried under vacuum, and the RuNC was recovered. The yields of the reactions progressively declined very insignificantly up to the fourth cycle and slightly more rapidly in the subsequent cycles. To understand the change in the nature of the catalyst after its recovery, we also subjected the recovered catalyst to TEM and TEM–SAED analysis (Figures S12 and S13, Supporting Information File 1) and found that it remained consistent with “fresh” RuNC.

### Homogeneous vs heterogeneous mechanism of catalysis

The actual nature of the catalytic species in metal nanoparticle-catalysed C–C bond formation reactions has been a matter of debate, with several studies pointing out that the actual reaction occurs on the surface of the nanocatalyst through a heterogeneous mechanism, while other groups provided evidence that the metal nanoparticles actually act as a reservoir for soluble metal species formed by leaching that are the actual catalytic species responsible for the activity through a homogeneous mechanism [53–58]. Nevertheless, it is very difficult to confidently establish the actual operative mechanism and species as well as the heterogeneity/homogeneity of the catalysis. Several experimental tests were proposed to establish these, but each had its own limitations. To elaborate the homogeneous/heterogeneous nature of the catalysis by the RuNC, we carried out some of the recommended and accepted tests. As a preliminary experiment, we employed the Hg poisoning test for the reaction between **1a** and **2a** using the solid catalyst as well as the as-synthesised dispersed RuNC solution. The reaction was initially carried out for 2 h under the standard conditions, after which about 20% of the starting material was converted to the product. Then, Hg was added to the reaction mixture, and the reaction was continued for further 10 h, at the end of which an analysis indicated that the addition of Hg had completely inhibited product formation [59].

**Scheme 3:**
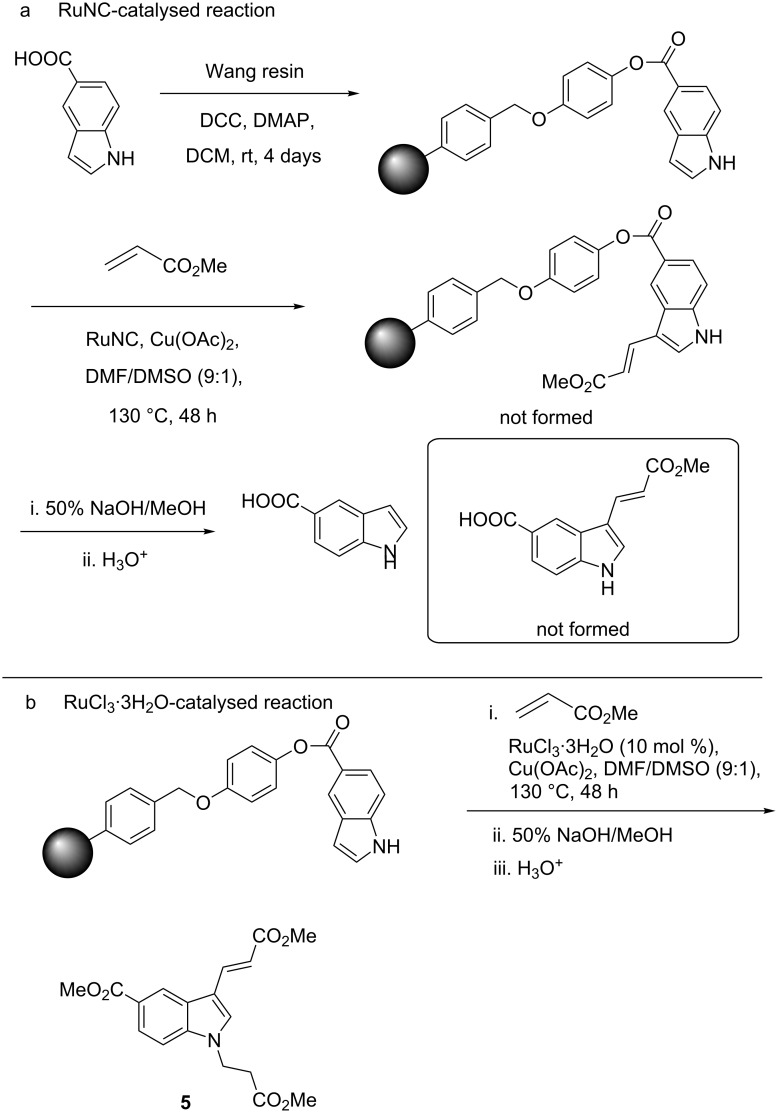
a) Three-phase test to determine a homogeneous or heterogeneous catalytic mechanism of action for the RuNC. b) Control experiment for the reaction of an anchored indole derivative under homogeneous catalysis with RuCl_3_⋅3H_2_O.

For further confirmation of the heterogeneous reaction pathway, we also carried out the three-phase test (Scheme 3) [60]. For this purpose, indole-5-carboxylic acid was anchored to Wang resin and then subjected to the conditions for the alkenylation reaction with **2a** using the solid catalyst (3 mg) for 48 h. After that, the reaction mixture was worked up, and the solid product was isolated and subjected to solid-state NMR spectroscopy. The results were then compared to, and found to be identical to, that for the indole-anchored Wang resin used as substrate for the reaction (Figures S16–S18, Supporting Information File 1). As a control experiment, the homogeneous alkenylation reaction of the Wang resin-anchored indole derivative was also carried out using a significantly higher loading of RuCl_3_ (10 mol %) under the optimised conditions for 48 h. Analysis of the product after the experiment by IR spectroscopy indicated the presence of an additional peak at 1610 cm^−1^ for a C=O moiety. For further confirmation of the alkenylation reaction, the solid product was hydrolysed with aqueous NaOH, and the reaction mixture was then acidified with aqueous HCl to yield the product **5**, which was characterised by spectroscopic techniques. The formation of the product **5** could be rationalised by the following: The C–H alkenylation reaction of the Wang resin-anchored indole-5-carboxylic acid was successful during the homogeneous two-phase alkenylation reaction. Subsequently, during its removal from the support under alkaline conditions, N*-*alkylation occurred through a Michael addition to the acrylate **2a**, followed by the formation of the methyl ester of the 5-carboxylic acid during the acidification of the reaction mixture in MeOH. These experiments established that the reaction was not taking place with any leached homogeneous Ru species within any detectable limits, and most certainly, the catalyst was acting through a heterogeneous mechanism.

Further proof for the heterogeneous mechanism was also found through ICP–MS studies of the reaction mixture between **1a** and **2a**. The ICP–MS analysis of the reaction mixture was carried out after removal of all the solids by centrifugation in the middle of the reaction. It showed that the content of Ru in the solution phase was negligible (2.1 ppb). While from these results, it may be possible that the actual catalyst for the reactions under the standard conditions was the leached homogeneous species of Ru, such as clusters [53–58,61–65], the ICP–MS results taken in conjunction with the results of the Hg poisoning test and, more importantly, the three-phase test, could allow us to reach the conclusion that the reactions were catalysed by a heterogeneous process.

### Role of the surface oxide and plausible mechanism

**Scheme 4:**
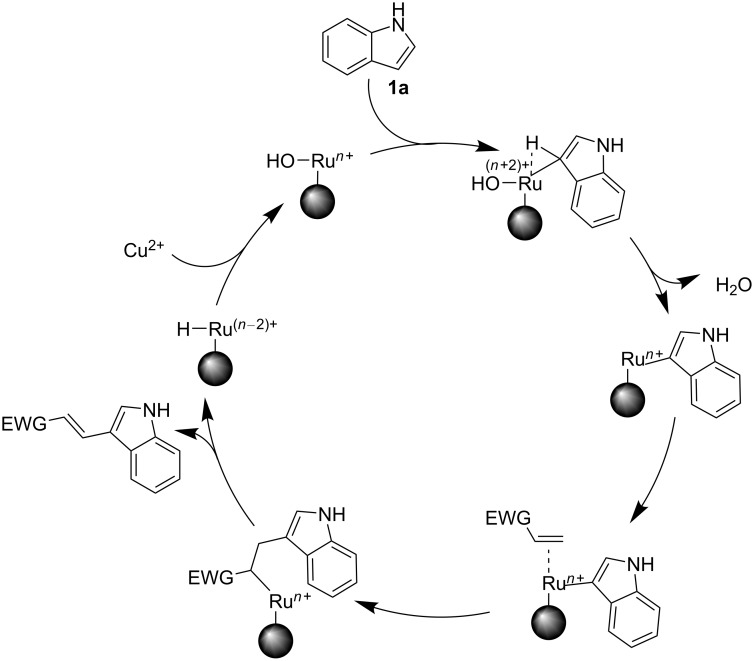
Probable catalytic mechanism for the transformation of **1a** by the RuNC.

One of the reasons for the high catalytic activity of the RuNC was the near-naked nature, since it is well established that Ru nanoparticles that lack stabilisers on their surface are catalytically more active than those with stabilisers [66]. The presence of surface oxides on the ruthenium nanoparticles is interesting: On the face of it, it is a digression from our initial target to synthesise zerovalent Ru nanoparticles. However, with respect to their catalytic ability, they are actually beneficial and responsible for the catalytic activity of the nanocatalyst towards the C–H activation reaction, since it has previously been shown that the presence of surface oxides on essentially zerovalent Ru nanoparticles promotes their catalytic ability towards several challenging reactions, such as CO oxidation [67–68] and hydrogen evolution [69]. Interestingly, pristine Ru(0) single crystals have been reported to perform poorly in these reactions when compared to the surface-oxidised ones [54,70]. With respect to C–H activation reactions, the presence of surface oxides on our RuNC probably governed its ability to catalyse the C–H alkenylation reaction, in contrast to the role of reduced Ru(0) nanoparticles with hydride/deuteride species on their surface, reported by Pieters et al. for C–H deuteration reactions occurring α to the nitrogen atom [43–44]. The synergistic effect of the surface oxides in promoting the efficiency of zerovalent Ru nanoparticles is also documented through experimental as well as computational results in a study of the C–H selenylation of indoles where the C–H activation reactions were initiated by the oxidised Ru species on the surface [45]. Again, strongly oxidising reaction conditions due to the presence of Cu(OAc)_2_ as the oxidant further attenuated the preservation and regeneration of the surface oxides following any catalytic cycle, which enabled the catalytic activity to be maintained for subsequent cycles. A probable mechanism for the synthesised RuNC is presented in Scheme 4.

## Conclusion

In conclusion, this work describes the C–H alkenylation of indoles **1** catalysed by colloidal Ru@RuO_x_ nanoparticles. The C–H alkenylation reaction tolerated several functional groups, including bromine and nitrile units, which provide ample scope for further manipulation of the products from the perspective of medicinal chemistry. The catalyst can be easily recovered and recycled in a colloidal solid form, enabling catalytic recycling and reusability. Mechanistic studies have unambiguously proven the heterogeneous nature of the catalysis. The ability of the nanocatalyst to activate the C–H bond is due to the presence of minimal stabilising groups on its surface. Studies of the surface morphology of the catalyst have revealed the presence of surface oxides RuO_x_ on the RuNC, which is responsible for the high catalytic activity in the C–H activation reaction.

## Supporting Information

File 1 Figures for the characterisation of the Ru nanocatalyst, detailed experimental procedures, and product characterisation data, along with ^1^H and ^13^C NMR spectra.
