# Supplementary material for: Synthesis of 3-alkenylindoles through regioselective C–H alkenylation of indoles by a ruthenium nanocatalyst
Source: Beilstein J Org Chem. 2020 Jan 29;16:140–8. doi: 10.3762/bjoc.16.16 (PMC7006491; doi:10.3762/bjoc.16.16)
Supplement: File 1 — Figures for the characterisation of the Ru nanocatalyst, detailed experimental procedures, and product characterisation data, along with 1H and 13C NMR spectra. [file Beilstein_J_Org_Chem-16-140-s001.pdf]

BEILSTEIN JOURNAL OF ORGANIC CHEMISTRY

Supporting Information

for

Synthesis of 3-alkenylindoles through regioselective C–H alkenylation of indoles by a ruthenium nanocatalyst

Abhijit Paul, Debnath Chatterjee, Srirupa Banerjee and Somnath Yadav

Beilstein J. Org. Chem. **2020,** *16,* 140–148. doi:10.3762/bjoc.16.16

Figures for the characterisation of the Ru nanocatalyst, detailed experimental procedures, and product characterisation data, along with ^{1}H and ^{13}C NMR spectra

License and Terms: This is a supporting information file under the terms of the Creative Commons Attribution License (https://creativecommons.org/licenses/by/4.0). Please note that the reuse, redistribution and reproduction in particular requires that the authors and source are credited.
The license is subject to the *Beilstein Journal of Organic Chemistry* terms and conditions: (https://www.beilstein-journals.org/bjoc)

Experimental

General procedures

The starting materials and all solvents used were purchased from commercial sources. Anhydrous solvents were prepared using standard methods. TLC was performed on precoated aluminium plates of silica gel 60 F_{254}. TLC spots were visualised by UV light (254 nm). Photolysis was carried out in an immersion well cooled by chilled water using a medium-pressure Hg vapour lamp. HRTEM was performed using a JEM-2100 HRTEM spectrometer model. XPS was performed with a PHI 5000 Versa Probe II, FEI Inc. The powder X-ray diffraction (p-XRD) experiment was performed on a Rigaku Smartlab X-ray diffractometer equipped with CuK_{α} radiation (λ = 0.15406 nm). ^{1}H and ^{13}C NMR spectra were recorded on a Bruker 400 MHz NMR device in solution of $CDCl_3$ or DMSO-d_6.

Procedure for the synthesis of the colloidal Ru nanocatalyst

A solution of (*E*)-3-(2-methoxynaphthalen-1-yl)-1-(4-methoxyphenyl)prop-2-en-1-one (95 mg, 0.3 mmol) and $RuCl_3{\cdot}3H_2O$ (21 mg, 0.1 mmol) in anhydrous MeOH (120 mL) was deaerated by bubbling argon for 30 minutes. After that, the solution was photolysed in an immersion well reactor with a 450 W medium pressure Hg vapour lamp for 30 minutes. The resulting solution of Ru nanoparticles was either characterised by TEM and HRTEM for the detection of the Ru nanoparticles. Additionally, centrifugation of the solution was also carried out at 17000 rpm to separate the Ru nanocatalyst for further characterisation and catalytic studies.

General procedure for the C3–H alkenylation of indoles

To a mixture of indole (**1**, 1 mmol), alkene **2** (2 mmol), and $Cu(OAc)_2$ (1.8 mmol) in anhydrous DMF/DMSO, 9:1 (5 mL), 3 mg of RuNC (3×10^{-5} mol) was added, and the resulting mixture was heated with stirring at 130 °C under Argon atmosphere for 12 h. After completion of the reaction (monitored by TLC analysis), the reaction mixture was cooled, diluted with ethyl acetate (EtOAc), and then filtered through celite-545. The EtOAc layer was then washed with brine, dried with anhydrous Na_2SO_4, and concentrated in vacuo. The residue was then subjected to column chromatography on silica gel (100–200 mesh) using ethyl acetate/petroleum ether (EtOAc/PE) as eluent to afford the desired 3-alkenylindoles **3**.

Synthesis of Wang resin-bound indole-5-carboxylate

Wang resin (0.625 g) was added to anhydrous DCM (7.5 mL) and allowed to stir at room temperature for 20 min to allow it to swell. Then, indole-5-carboxylic acid (0.452 g, 2.81 mmol), DCC (0.580 g, 2.81 mmol), and DMAP (0.069 g, 0.565 mmol) were added to the swelled Wang resin, and the mixture was stirred for 4 days. After TLC indicated the complete disappearance of indole-5-carboxylic acid, the solid material was filtered and washed successively three times each with 5 mL of DMF, THF, MeOH, and finally with DCM. The resin-bound material was then dried in vacuo for 24 hours, and the resin-bound indole derivative was then characterised by FTIR and solid-state NMR spectroscopy.

Procedure for the three-phase test of the C–H alkenylation of indoles

To a mixture of resin-bound indole-2-carboxylate (250 mg), methyl acrylate (2 mmol), $Cu(OAc)_2$ (1.8 equiv), and DMF/DMSO, 9:1, v/v (10 mL), 3 mg of RuNC was added, and the resulting reaction mixture was heated at 130 °C for 48 h under argon atmosphere. Then, the reaction mixture was filtered and the residue washed three times each with EtOAc, DCM, and MeOH, after which it was dried under vacuum and characterised by FTIR spectroscopy. The IR spectrum was identical to that of the starting material, indicating that no 3-alkenyl derivative had formed. Subsequently, the hydrolysis of the indole moiety from the resin support was carried out. For this, the resin-bound material obtained after the attempted reaction (150 mg) was hydrolysed with aqueous NaOH (50%) and filtered to obtain a clear solution. Then, the solution was acidified with 6 N HCl to afford an off-white solid compound, which was characterised by spectral analysis as pure indole-5-carboxylic acid (by superimposing the FTIR spectrum with an authentic sample of indole-5-carboxylic acid [8], see Figure S15). mp 210 °C; ^{1}H NMR (400 MHz, DMSO-d_6): δ 6.58 (t, *J* = 2 Hz, 1H), 7.46 (dd, *J* = 2.8; 5.4 Hz, 2H), 7.72 (dd, *J* = 1.6; 8.6 Hz, 1H), 7.63 (d, *J* = 8.0 Hz, 1H), 8.25 (s, 1H), 11.42 (s, 1H), 12.35 (s, 1H); ^{13}C NMR (100 MHz, DMSO-d_6) δ 103.0, 111.6, 121.9, 122.7, 123.3, 127.4, 127.7, 138.8, 168.9.

Procedure for the homogeneous C–H alkenylation of anchored indole with $RuCl_3{\cdot}3H_2O$

Wang resin-anchored indole-5-carboxylic acid was reacted with **2a** under conditions similar to those of the three-phase test described above in the presence of $RuCl_3{\cdot}3H_2O$ (20 mg, 10 mol %). After the reaction, the solid product was isolated, hydrolysed and then

acidified as described previously. Subsequent, chromatographic separation of the crude product led to the isolation of 27 mg of a product characterised as methyl 1-(2-(methoxycarbonyl)ethyl)-3-((*E*)-2-(methoxycarbonyl)vinyl)-1*H*-indole-5-carboxylate (**5**, 27 mg) as colourless oil. ^{1}H NMR (400 MHz, DMSO-d_6) δ 8.44 (s, 1H), 8.12 (s, 1H), 7.88–7.82 (m, 2H), 7.73 (d, *J* = 8.7 Hz, 1H), 6.36 (d, *J* = 16.0 Hz, 2H), 4.49 (t, *J* = 6.7 Hz, 2H), 3.87 (s, 3H), 3.71 (s, 3H), 3.55 (s, 3H), 2.91 (t, *J* = 6.7 Hz, 2H); ^{13}C NMR (100 MHz, DMSO-d_6) δ 171.6, 167.8, 167.3, 139.9, 138.0, 136.1, 125.6, 123.9, 123.0, 122.2, 112.8, 112.4, 111.7, 52.5, 52.1, 51.7, 42.4, 34.3; HRMS (*m*/*z*): [M + Na]$^{+}$ calcd for $C_{18}H_{18}NO_6Na$, 346.1285; found, 346.1287; IR (KBr) $\tilde{\nu}_{max}$: 1745, 1708, 1633 cm^{-1}.

Characterisation of compounds 3

3-(1*H*-Indol-3-yl)acrylic acid methyl ester [1] (3a)

Yellow solid; Yield: 163 mg, 81%; M. P: 150-152 °C; ^{1}H NMR (400 MHz, DMSO-d_6) δ 11.43 (bs, 1H), 7.85 (d, *J* = 16 Hz, 1H), 7.78 (d, *J* = 7.2 Hz, 1H), 7.60 (d, *J* = 2.5 Hz, 1H), 7.41 (d, *J* = 7 Hz, 1H), 7.14 (ddd, *J* = 14.3, Hz, *J* = 7.1 Hz and *J* = 5.6 Hz, 2H), 6.32 (d, *J* = 16 Hz, 1H), 3.71 (s, 3H); ^{13}C-NMR (100 MHz, DMSO-d_6) δ 166.7, 137.5, 136.2, 129.3, 123.5, 121.1, 119.5, 118.4, 111.0, 110.6, 109.7, 49.6.

3-(1*H*-Indol-3-yl)acrylic acid *tert*-butyl ester [2] (3b)

White solid; Yield: 183 mg, 76%; M.P: 123-125 °C; ^{1}H NMR (400 MHz, DMSO-d_6) δ 11.64 (s, 1H), 7.79 (t, *J* = 12.5 Hz, 3H), 7.45 (d, *J* = 7.3 Hz, 1H), 7.21-7.13 (m, 2H), 6.27 (d, *J* = 16 Hz, 1H), 1.51 (s, 9H); ^{13}C-NMR (100 MHz, DMSO-d_6) δ 166.7, 137.7, 137.3, 130.7, 125.0, 122.2, 120.6, 119.6, 113.0, 112.2, 111.6, 28.0.

3-(5-Methoxy-1*H*-indol-3-yl)acrylic acid methyl ester [1] (3c)

Brown oil; Yield: 178 mg, 77%; ^{1}H NMR (400 MHz, DMSO-d_6) δ 11.66 (s, 1H), 7.90 (dd, *J* = 9.3 Hz and 6.4 Hz, 2H), 7.37 (d, *J* = 8.8 Hz, 1H), 7.29 (d, *J* = 1.8 Hz, 1H), 6.85 (dd, *J* = 8.8 Hz and *J* = 2.2 Hz, 1H), 6.33 (d, *J* = 16 Hz, 1H), 3.82 (s, 3H), 3.70 (s, 3H); ^{13}C-NMR (100 MHz, DMSO-d_6) δ 167.8, 154.8, 139.0, 132.2, 131.8, 125.6, 113.1, 112.4, 111.5, 110.1, 101.7, 55.5, 50.9.

3-(5-Methoxy-1*H*-indol-3-yl)acrylic acid *tert*-butyl ester (3d)

Yellow oil; Yield: 202 mg, 74%; ^{1}H NMR (400 MHz, DMSO-d_6) δ 11.52 (s, 1H), 7.76 (d, *J*= 5.4 Hz, 1H), 7.74 (d, *J*= 7.6 Hz, 1H), 7.34 (d, *J* = 8.8 Hz, 1H), 7.21 (d, *J* = 2.3 Hz, 1H), 6.83 (dd, *J* = 8.8 Hz and *J* = 2.3 Hz, 1H), 6.18 (d, *J*= 16 Hz, 1H), 3.83 (s, 3H), 1.51 (s, 9H); ^{13}C-NMR (100 MHz, DMSO-d_6) δ 166.8, 154.6, 137.7, 132.2, 130.8, 125.6, 112.8, 112.4, 112.0, 111.4, 101.6, 55.4, 28.0. HRMS calcd. for $C_{16}H_{19}NO_3Na^+$, 296.1263, found, 296.1265.UV (MeOH) :λ_{max}(log ε) 331 nm (7.25), 281.5 nm (7.08), 226.5 nm (7.23). IR (ATR, ṽ cm^{-1}): 3291, 1666, 1619.

3-(5-Bromo-1*H*-indol-3-yl)acrylic acid methyl ester [1] (3e)

Brown oil; Yield: 203 mg, 73%; ^{1}H NMR (400 MHz, DMSO-d_6) 11.93 (s, 1H), 8.01 (dd, *J* = 2.8 Hz and 9.2 Hz, 2H), 7.85 (d, *J* = 16 Hz, 1H), 7.43 (d, *J* = 8.8 Hz, 1H), 7.32 (dd, *J* = 2 Hz and 8.6 Hz, 1H), 6.37 (d, *J* = 16.4 Hz, 1H), 3.70 (s, 3H); ^{13}C-NMR (100 MHz, DMSO-d_6) 167.5, 138.1, 136.0, 132.5, 126.7, 125.0, 121.9, 114.3, 113.6, 111.5, 111.2, 51.0.

3-(5-Bromo-1*H*-indol-3-yl)acrylic acid *tert*-butyl ester (3f)

Red oil; Yield: 250 mg, 78%; ^{1}H NMR (400 MHz, $CDCl_3$) 8.67 (s, 1H), 8.02 (s, 1H), 7.73 (d, *J* = 16 Hz, 1H), 7.42 (d, *J* = 2.4 Hz, 1H), 7.34 (d, *J* = 8.8 Hz, 1H), 7.26 (d, *J* = 8.8 Hz, 1H), 6.32 (d, *J* = 16 Hz, 1H), 1.55 (s, 9H); ^{13}C-NMR (100 MHz, $CDCl_3$) 167.5, 136.3, 135.7, 129.1, 127.0, 126.2, 123.1, 116.2, 114.7, 113.3, 113.1, 80.2, 28.3. HRMS calcd. for $C_{15}H_{16}BrNO_2Na^+$, 344.0262, found, 344.0265.UV (MeOH): λ_{max}(log ε) 323.5 nm (5.25), 282.5 nm (5.15), 230.5 nm (5.48). IR (KBr, ṽ cm^{-1}): 3220, 1672, 1609.

3-(5-Cyano-1*H*-indol-3-yl)acrylic acid methyl ester [3] (3g)

Grey solid; Yield: 201 mg, 89%; M.P: 134-135 °C; ^{1}H NMR (400 MHz, DMSO-d_6) δ 12.23 (s, 1H), 8.50 (s, 1H), 8.15 (s, 1H), 7.89 (d, *J* = 8.44 Hz, 1H), 7.55 (dd, *J* = 8.44 Hz and 1.44 Hz, 2H), 6.55 (d, *J* = 16.1 Hz, 1H), 3.72 (s, 3H); ^{13}C-NMR (100 MHz, DMSO-*d*6) δ 168.0, 139.5, 137.9, 133.7, 125.8, 125.8, 125.3, 120.7, 113.4, 112.8, 103.5, 51.5. HRMS calcd. for $C_{13}H_{10}N_2O_2Na^+$, 249.0639, found, 249.0633.UV (MeOH) :λ_{max}(log ε) 331 nm (7.38), 289 nm (7.29), 243 nm (7.60). IR (KBr, ṽ cm^{-1}): 3449, 2225, 1718, 1632.

3-(5-Cyano-1*H*-indol-3-yl)acrylic acid *tert*-butyl ester [4] (3h)

Brown solid; Yield: 225 mg, 84%; M.P: 108-110 °C; ^{1}H NMR (400 MHz, DMSO-*d*6) δ 8.24 (s, 1H); 7.76 (d, *J* = 16.1 Hz, 1H), 7.57 (d, *J* = 2.3 Hz, 1H), 7.50 (s, 2H), 6.38 (d, *J* = 16.1 Hz, 1H), 1.56 (s, 9H); ^{13}C-NMR (100 MHz, DMSO-*d*6) δ 167.1. 138.8, 135.4, 129.6, 126.0, 125.3, 120.2, 117.5, 112.7, 114.2, 80.5, 28.3. HRMS calcd. for $C_{16}H_{16}N_2O_2Na^+$, 291.1109, found, 291.1105.UV (MeOH): λ_{max}(log ε) 329 nm (6.95), 289 nm (6.88), 241.5 (7.26). IR (KBr, ṽ cm^{-1}): 3474, 2231, 1705, 1635.

3-(2-(Methoxycarbonyl)vinyl)-1*H*-indole-2-carboxylic acid ethyl ester [5] (3i)

White solid; Yield: 229 mg, 84%; M.P: 171-173 °C; ^{1}H NMR (400 MHz, DMSO-*d*6) δ 12.25 (s, 1H), 8.61 (d, *J* = 16.3 Hz, 1H), 7.95 (d, *J* = 8.2 Hz, 1H), 7.56 (d, *J* = 8.2 Hz, 1H), 7.32-7.38 (m, 1H), 7.22 (t, *J* = 7.4 Hz, 1H), 6.60 (d, *J* = 16.4 Hz, 1H), 4.43 (q, *J* = 7.2 Hz, 2H), 3.77 (s, 3H), 1.40-1.56 (m, 3H); ^{13}C-NMR (100 MHz, DMSO-*d*6) δ 165.1, 159.1, 135.9, 135.0, 125.5, 123.5, 123.1, 120.2, 119.7, 114.9, 113.8, 111.6, 59.2, 49.4, 12.4.

3-(2-(*tert*-Butoxycarbonyl)vinyl)-*1H*-indole-2-carboxylic acid ethyl ester [6] (3j)

Yellow solid; Yield: 277 mg, 88%; M.P: 143-145 °C;^{1}H NMR (400 MHz, $CDCl_3$) 9.22 (s, 1H), 8.53 (d, *J* = 16.3 Hz, 1H), 8.05 (d, *J* = 8.2 Hz, 1H), 7.46 (d, *J*= 8.2 Hz, 1H), 7.40 (t, *J* = 7.5 Hz, 1H), 7.28 (d, *J* = 14.4 Hz, 1H), 6.64 (d, *J* = 16.3, 1H), 4.51 (q, *J* = 7.1 Hz, 2H), 1.59 (s, 9H), 1.51 (t, *J* = 7.1 Hz, 3H); ^{13}C-NMR (100 MHz, $CDCl_3$) 166.8, 161.7, 136.1, 126.6, 126.0, 122.4, 122.1, 121.4, 117.3, 112.2, 61.7, 80.2, 28.3, 14.4. HRMS calcd. for $C_{18}H_{21}NO_4Na^+$, 338.1363, found, 338.1362.UV (MeOH): λ_{max}(log ε) 345 nm (6.98), 274 nm (7.03), 237 nm (6.99). IR (ATR, ṽ cm^{-1}): 3302, 1680, 1614.

3-(1-Methyl-1*H*-indol-3-yl)acrylic acid methyl ester [1] (3k)

Brown solid; Yield: 144 mg, 67%; M.P: 95-97 °C; ^{1}H NMR (400 MHz, DMSO-*d*6) 7.91 (s, 1H), 7.88 (d, *J* = 7.6 Hz, 1H), 7.84 (d, *J* = 16 Hz, 1H), 7.51 (d, *J* = 8 Hz, 1H), 7.27 (t, *J* = 7.2 Hz, 1H), 7.20 (t, *J* = 7.2 Hz, 1H), 6.35 (d, *J* = 16 Hz, 1H), 3.80 (s, 3H), 3.70 (s, 3H); ^{13}C-NMR (100 MHz, DMSO-*d*6) 168.2, 138.9, 138.3, 135.8, 125.9, 123.1, 121.7, 120.5, 111.2, 111.1, 111.1, 51.5, 33.4.

3-(1-Methyl-1*H*-indol-3-yl)acrylic acid *tert*-butyl ester [7] (3l)

Yellow oil; Yield: 180 mg, 70%; ^{1}H NMR (400 MHz, $CDCl_3$) 7.87 (d, *J* = 8 Hz, 1H), 7.75 (d, *J* = 16 Hz, 1H), 7.20-7.28 (m, 4H), 6.31 (d, *J* = 16 Hz, 1H), 3.75 (s, 3H), 1.51 (s, 9H); ^{13}C-NMR (100 MHz, $CDCl_3$) 167.8, 138.0, 136.9, 132.7, 126.1, 122.8, 121.1, 120.7, 114.6, 112.2, 109.9, 79.7, 33.1, 28.4.

3-(1-Benzyl-1*H*-indol-3-yl)acrylic acid methyl ester [1] (3m)

Colourless oil; Yield: 246 mg, 74%; ^{1}H NMR (400 MHz, DMSO-*d*6) 8.14 (s, 1H), 7.88 (t, *J* = 11 Hz, 2H), 7.54 (d, *J* = 8 Hz, 1H), 7.18-7.32 (m, 7H), 6.40 (d, *J* = 16 Hz, 1H), 5.45 (s, 2H), 3.70 (s, 3H); ^{13}C-NMR (100 MHz, DMSO-*d*6) 168.1, 138.8, 137.7, 137.6, 135.2, 129.1, 128.1, 127.6, 126.1, 123.2, 121.8, 120.7, 111.7, 111.6, 51.5, 49.9.

3-(1-Benzyl-1*H*-indol-3-yl)acrylic acid *tert*-butyl ester (3n)

Yellow oil; Yield: 221 mg, 76%; ^{1}H NMR (400 MHz, $CDCl_3$) 7.93 (dd, *J* = 6 Hz and 2.8 Hz, 1H), 7.80 (d, *J* = 16 Hz, 1H), 7.37 (s, 1H), 7.30 (d, *J* = 6.8 Hz, 4H), 7.24 (dd, *J* = 6.2 Hz and 3.2 Hz, 2H), 7.13 (d, *J* = 7.2 Hz, 2H), 6.37 (d, *J* = 15.6 Hz, 1H), 5.30 (s, 2H), 1.54 (s, 9H); ^{13}C-NMR (100 MHz, $CDCl_3$) 167.6, 137.6, 136.8, 136.3, 132.0, 129.0, 128.0, 127.0, 126.4, 123.0, 121.3, 120.7, 115.2, 112.8, 110.4, 79.8, 50.4, 28.4. HRMS calcd. for $C_{22}H_{23}NO_2Na^+$, 356.1626, found, 356.1621.UV (MeOH): λ_{max}(log ε) 332.5 nm (5.92), 274.5 nm (5.56), 226.5 nm (5.86), 202.5 nm (7.05). IR (KBr, ṽ cm^{-1}): 2970, 1694, 1620.

Methyl 1-(2-(methoxycarbonyl)ethyl)-3-((*E*)-2-(methoxycarbonyl)vinyl)-1*H*-indole-5-carboxylate (5)

Colourless oil; yield: 20 mg; ^{1}H NMR (400 MHz, DMSO-*d*6) δ 8.44 (s, 1H), 8.12 (s, 1H), 7.88 – 7.82 (m, 2H), 7.73 (d, *J* = 8.7 Hz, 1H), 6.36 (d, *J* = 16.0 Hz, 2H), 4.49 (t, *J* = 6.7 Hz, 2H), 3.87 (s, 3H), 3.71 (s, 3H), 3.55 (s, 3H), 2.91 (t, *J* = 6.7 Hz, 2H).; ^{13}C-NMR (100 MHz, DMSO-*d*6) δ 171.6, 167.8, 167.3, 139.9, 138.0, 136.1, 125.6, 123.9, 123.0, 122.2, 112.8, 112.4, 111.7, 52.5, 52.1, 51.7, 42.4, 34.3. HRMS calcd. for $C_{18}H_{18}NO_6H^+$, 345.1285, found, 346.1287. IR (KBr, ṽ cm-^{1}): 1745, 1708, 1633.

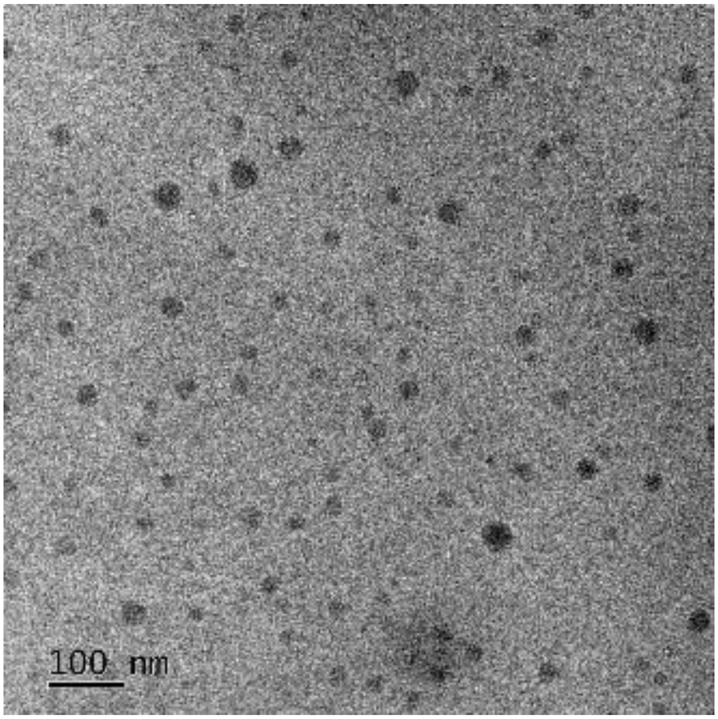

Figure S1: TEM of the as-synthesised RuNC.

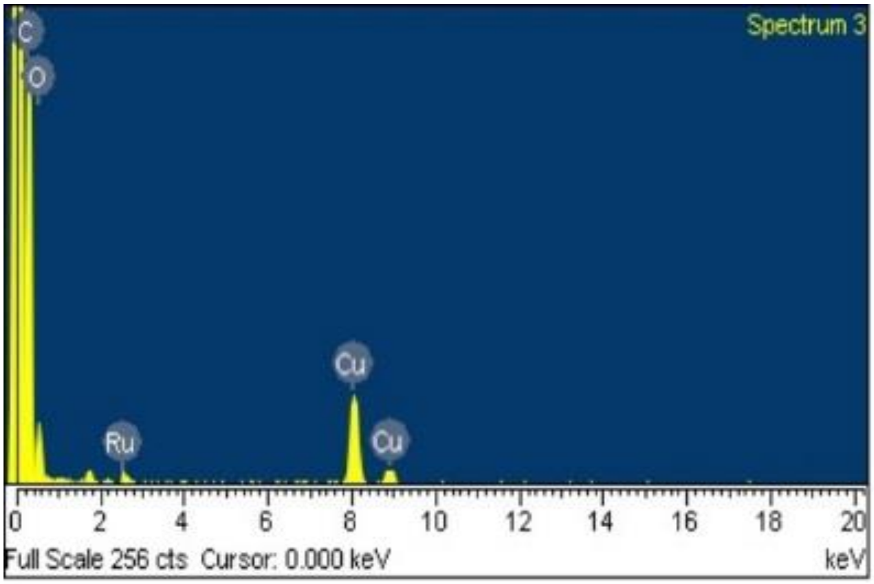

Figure S2: TEM–EDX of the as-synthesised RuNC.

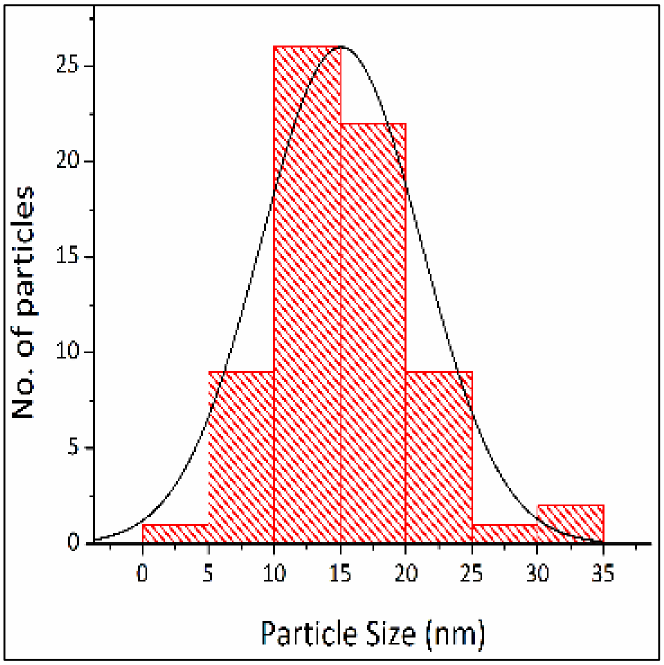

Figure S3: Particle size distribution of as-synthesised RuNC.

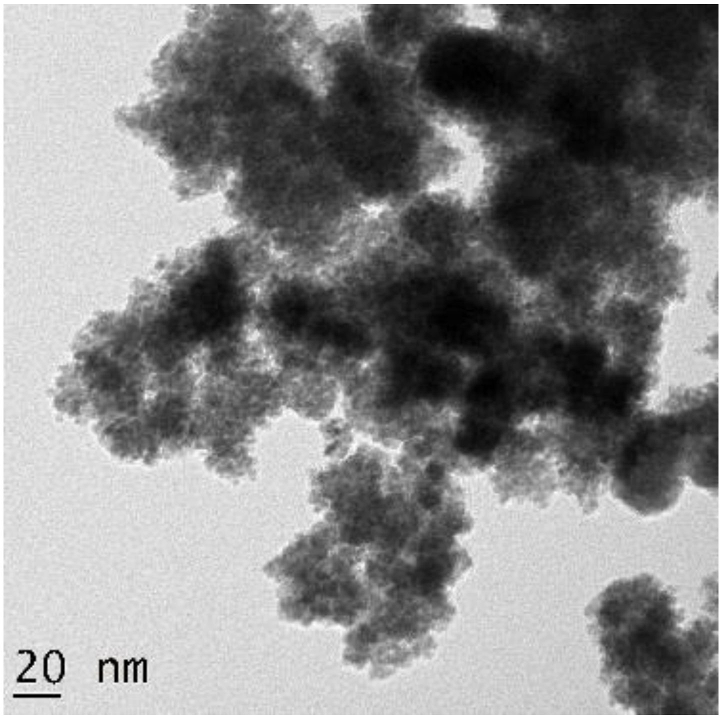

Figure S4: TEM image of the isolated and redispersed RuNC.

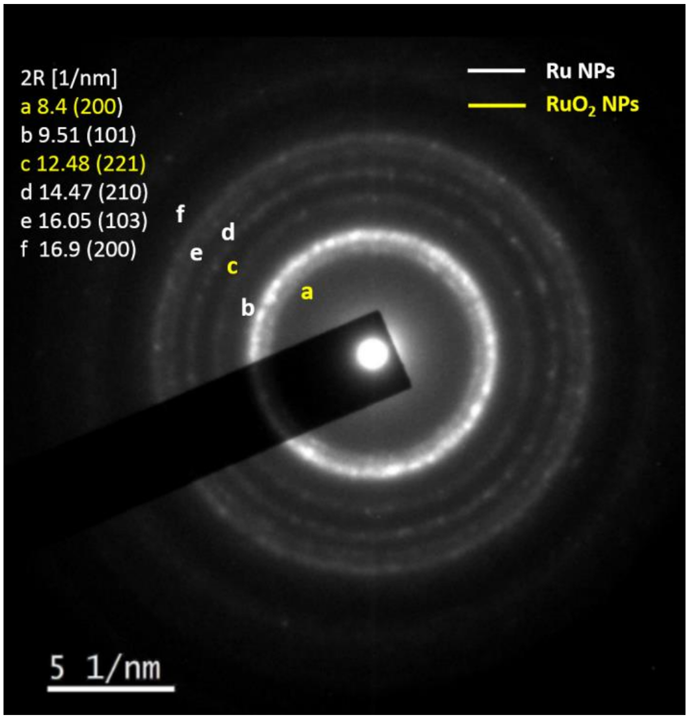

Figure S5: SAED image of the isolated and redispersed RuNC.

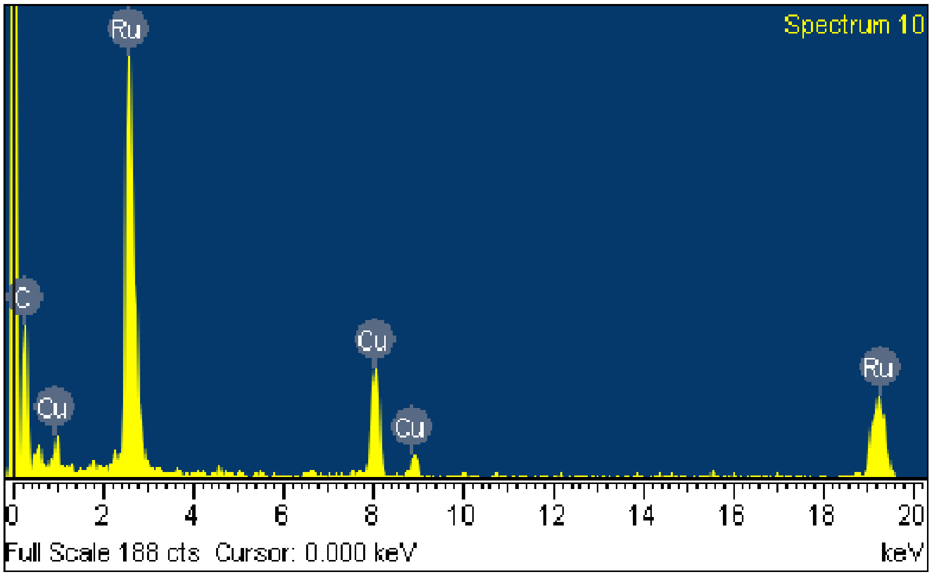

Figure S6: TEM–EDX image of the isolated and redispersed RuNC.

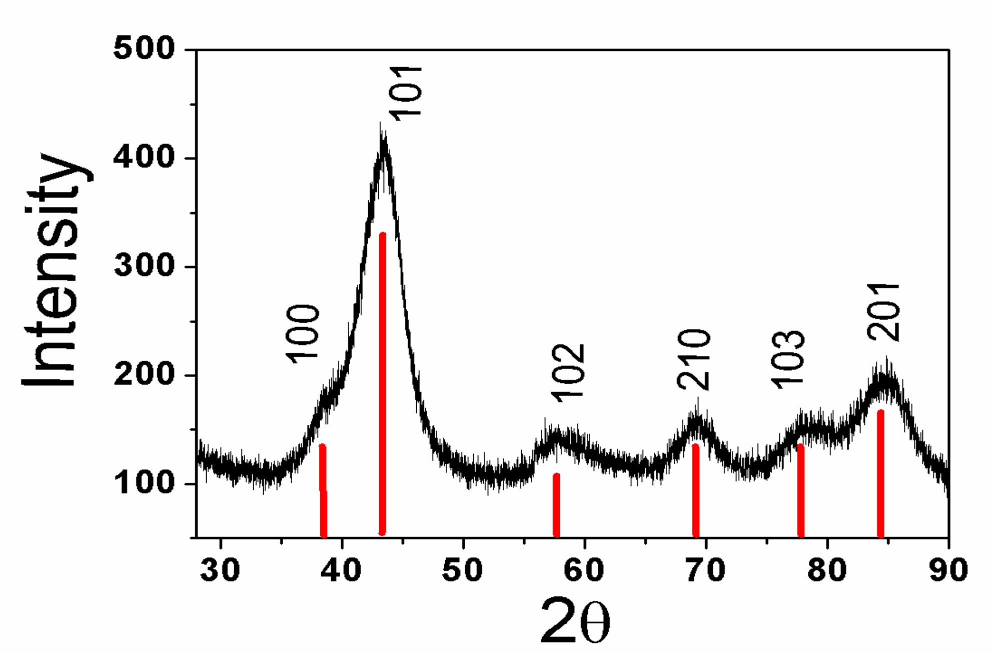

Figure S7: Powder XRD of the isolated RuNC.

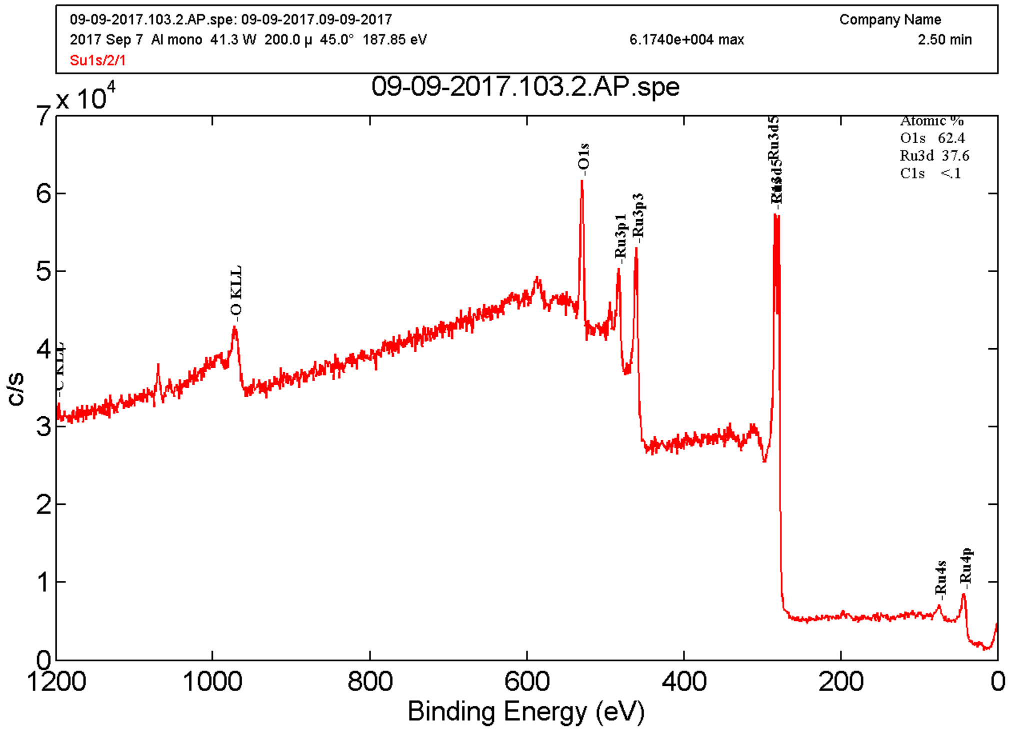

Figure S8: XPS spectrum of the RuNPs (survey scan).

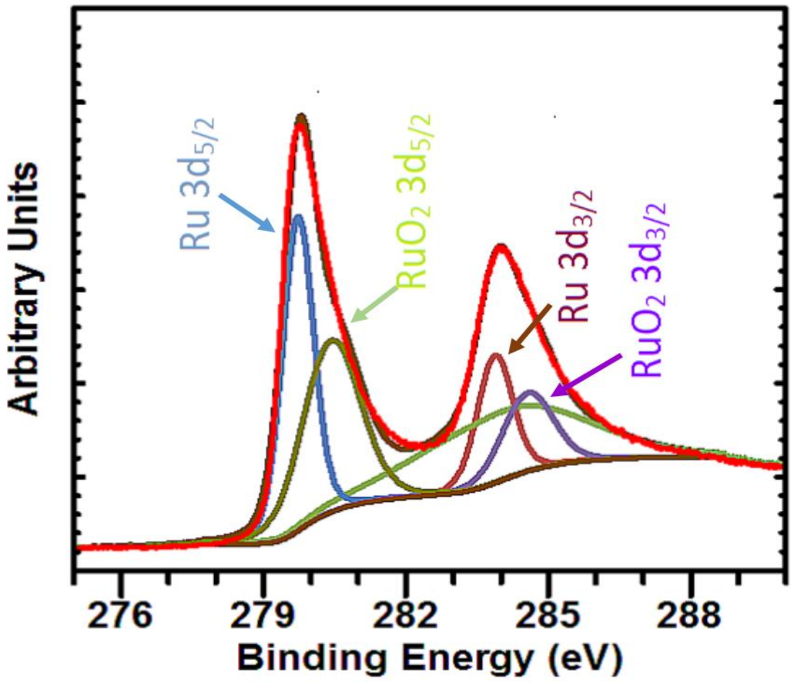

Figure S9: Deconvoluted XPS spectra of the RuNC (3d region).

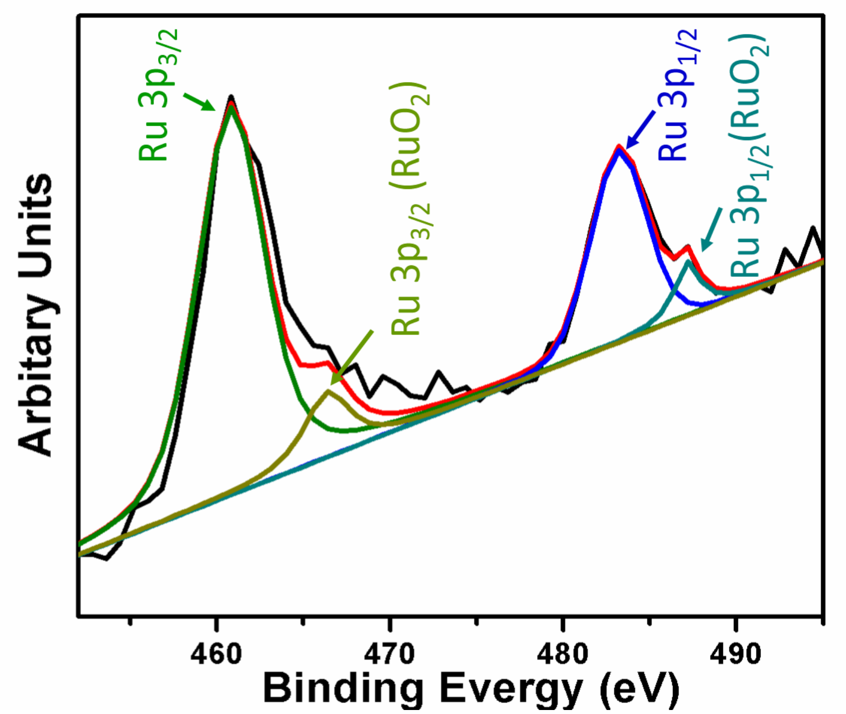

Figure S10: Deconvoluted XPS spectra of the RuNC (3p region).

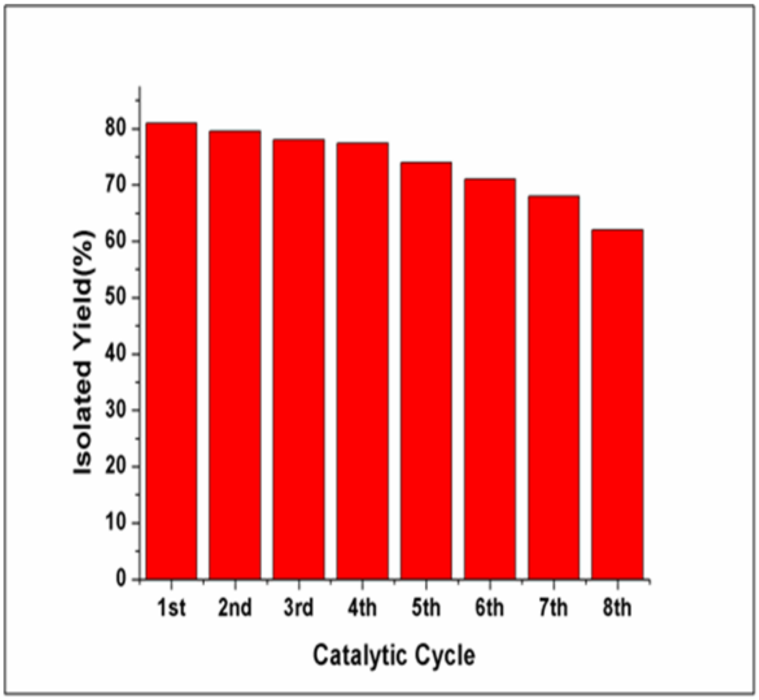

Figure S11: Recyclability of the RuNC for the C–H alkenylation reaction.

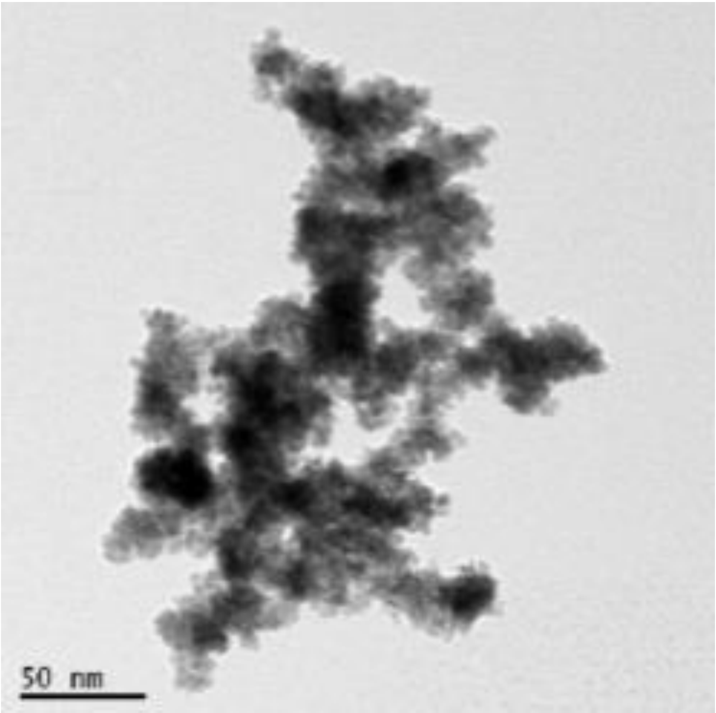

Figure S12: TEM image of the recovered RuNC after the 7th cycle.

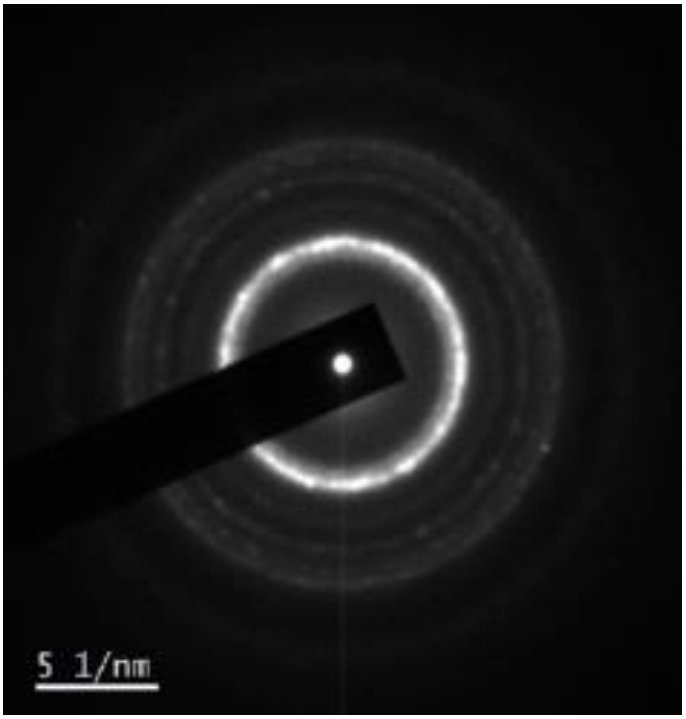

Figure S13: SAED image of the recovered RuNC after 7th cycle.

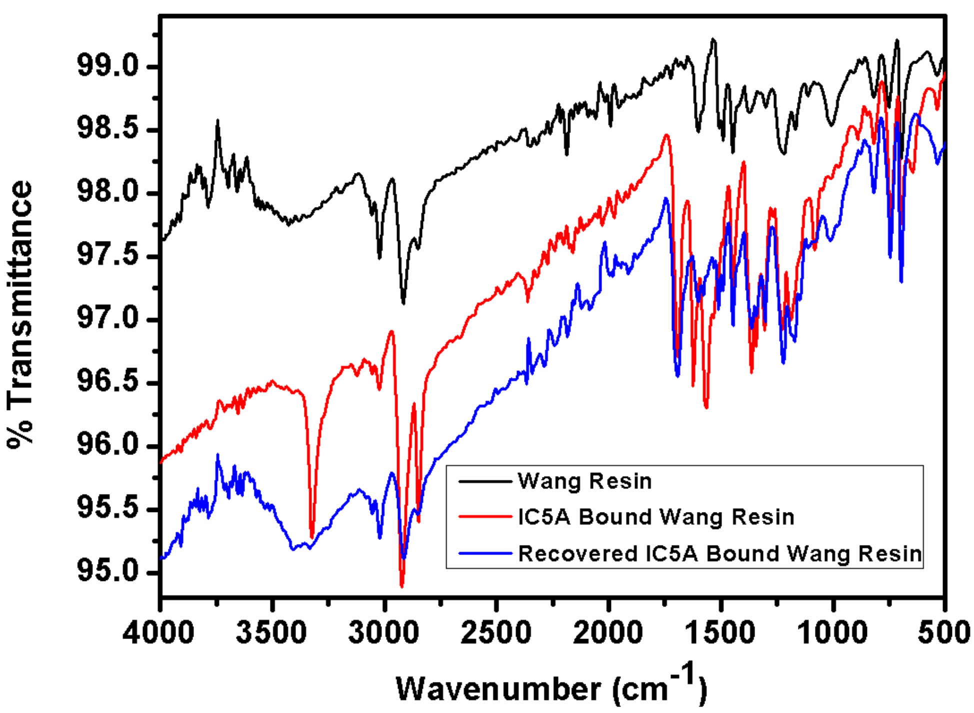

Figure S14: IR spectrum of pure Wang resin (black), indole-5-carboxylic acid-anchored Wang resin (red), and Wang resin-supported product of the three-phase test (blue).

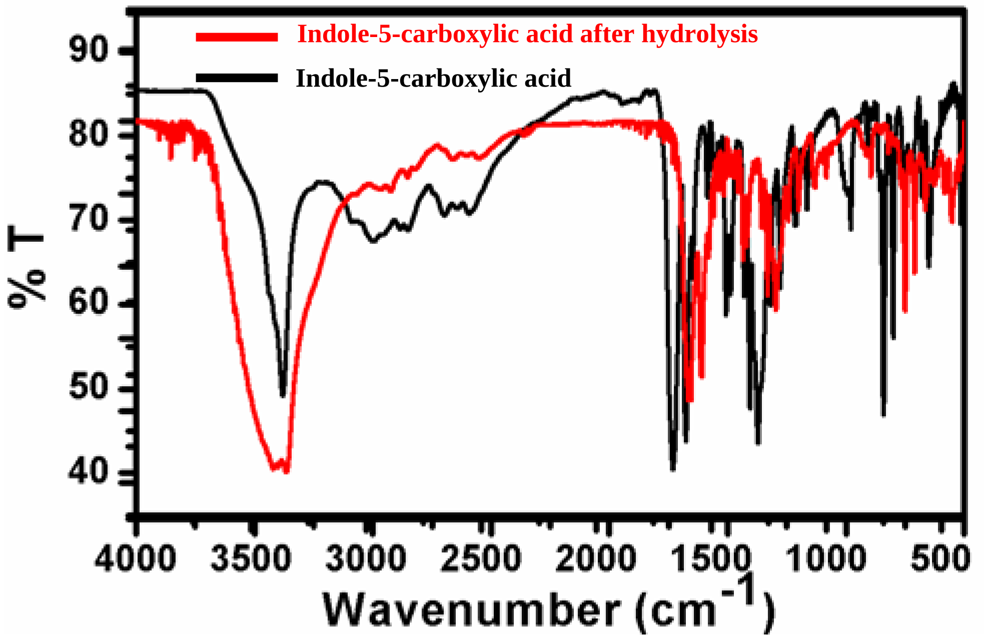

Figure S15: Superimposed FTIR spectra of pure indole-5-carboxylic acid and recovered material from the three-phase test.

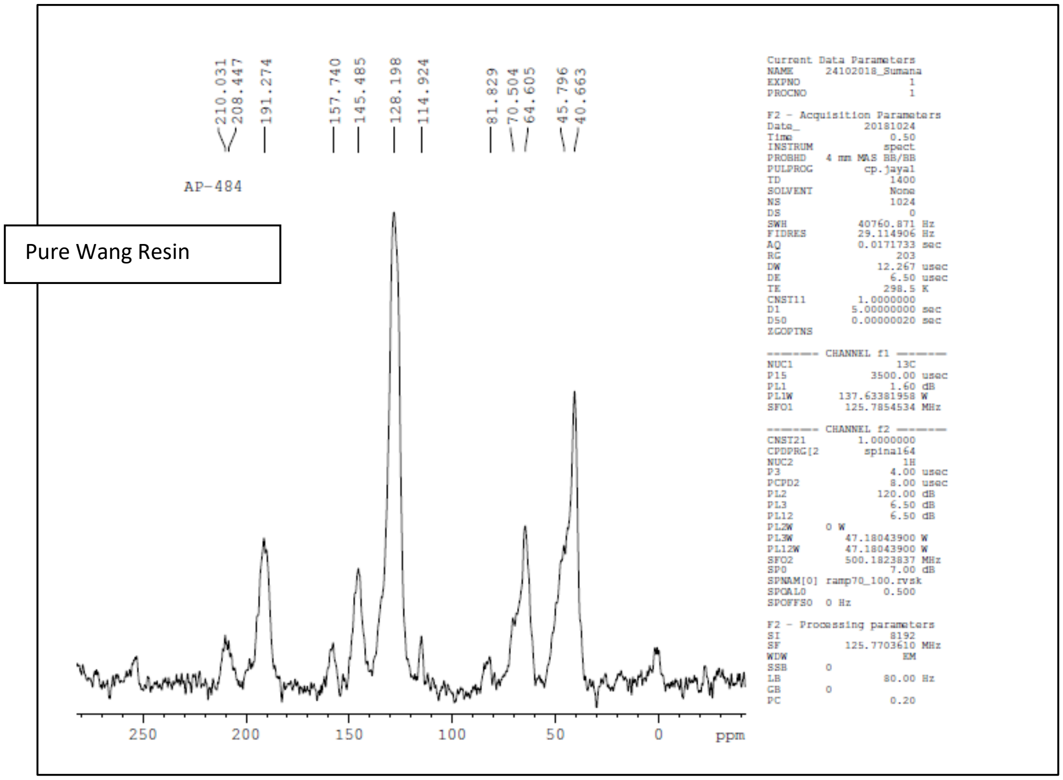

Figure S16: Solid-state ^{13}C NMR spectrum of pure Wang resin.

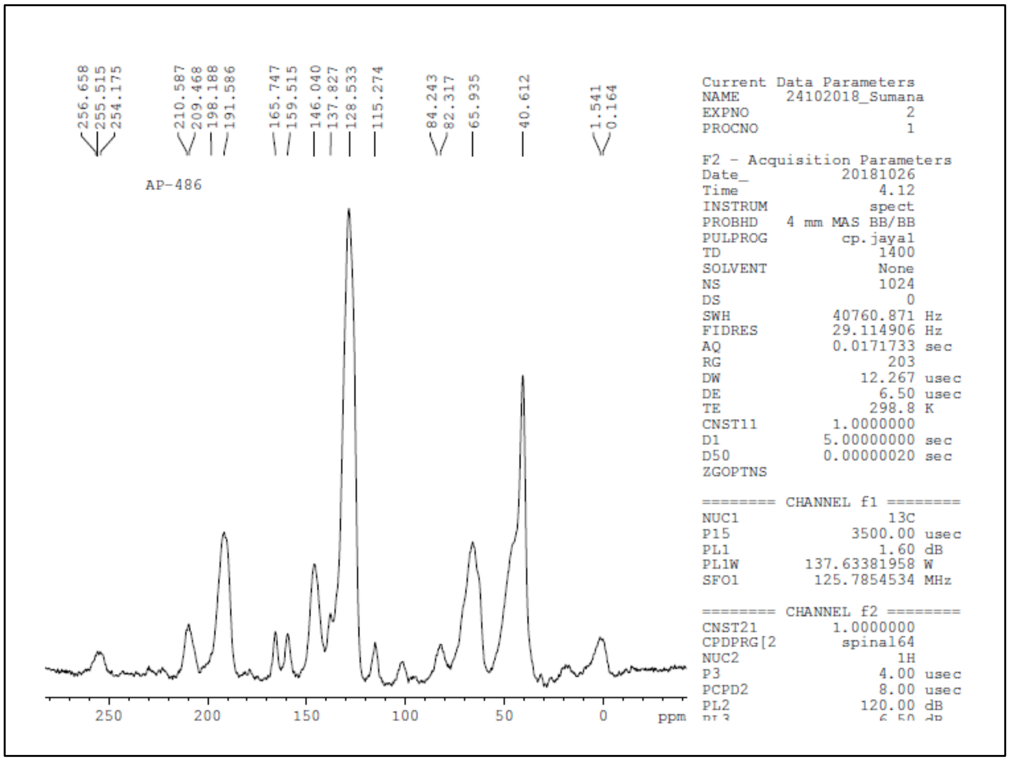

Figure S17: Solid-state ^{13}C NMR spectrum of indole-5-carboxylic acid-anchored Wang resin.

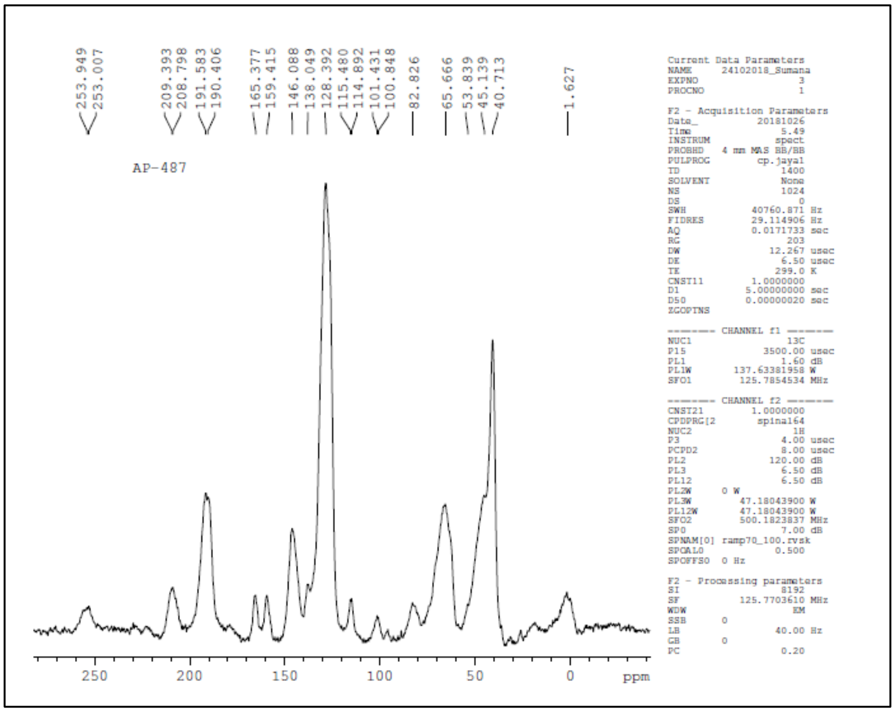

Figure S18: Solid-state NMR of indole-5-carboxylic acid-anchored Wang resin after C–H alkenylation.

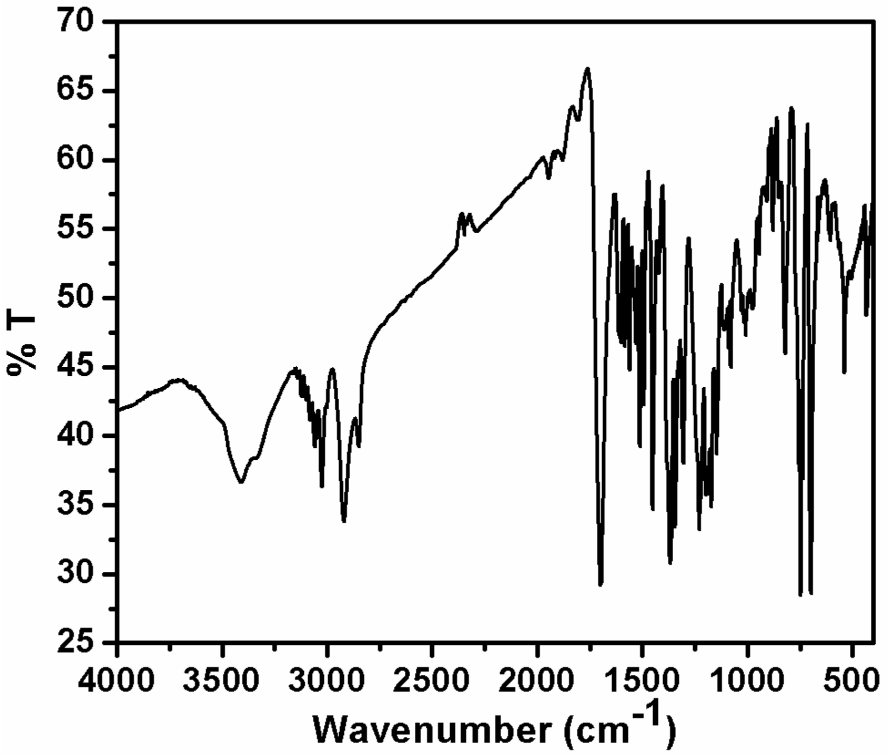

Figure S19: IR spectrum of indole-5-carboxyllic acid-anchored Wang resin after alkenylation with $RuCl_3$.

^{1}H and ^{13}C NMR Spectra

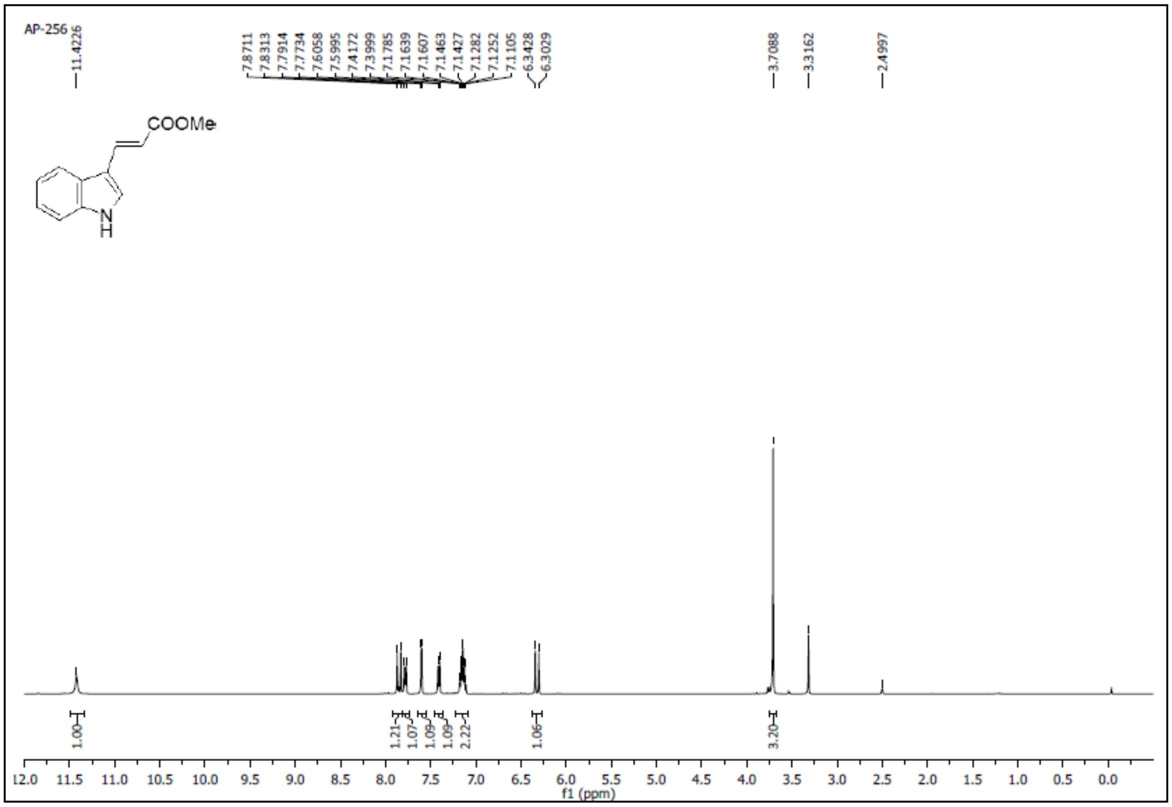

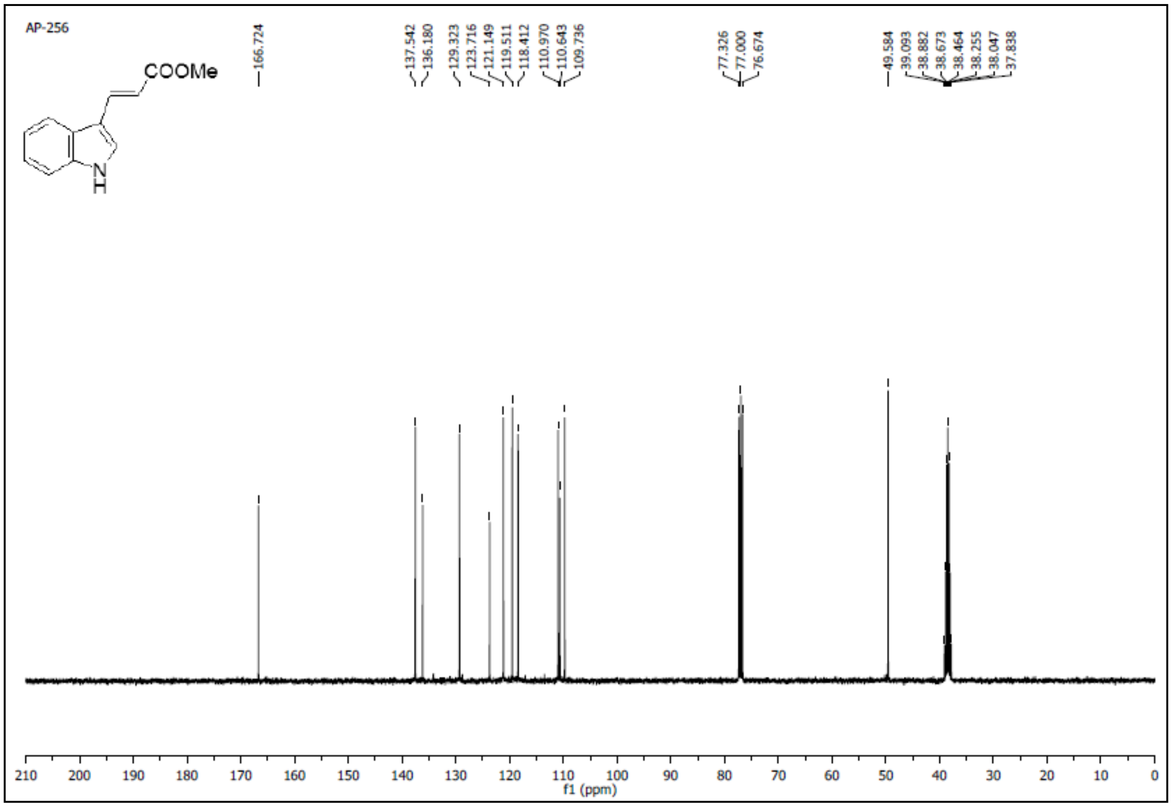

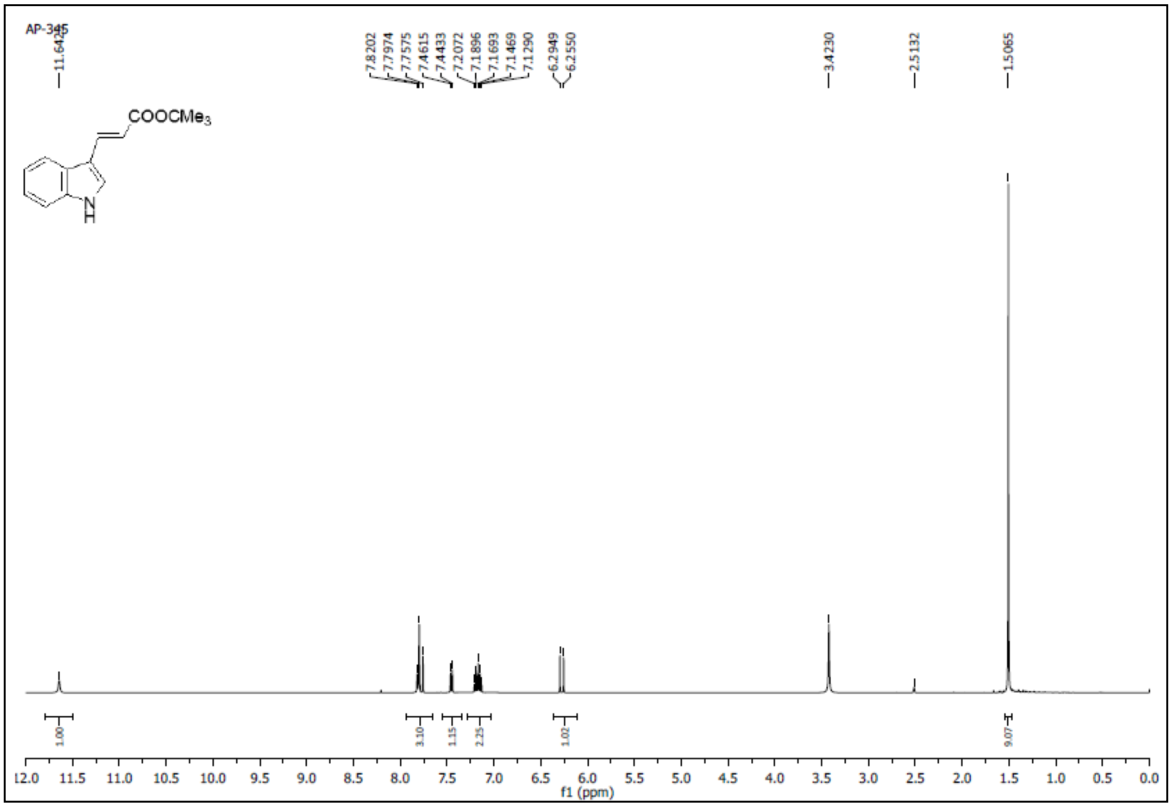

AP-345
11.6425
7.8202
7.7974
7.7575
7.4615
7.4433
7.2072
7.1896
7.1693
7.1469
7.1290
6.2949
6.2550
3.4230
2.5132
1.5065
COOCMe3
N
H
1.00
3.10
1.15
2.25
1.02
9.07
f1 (ppm)

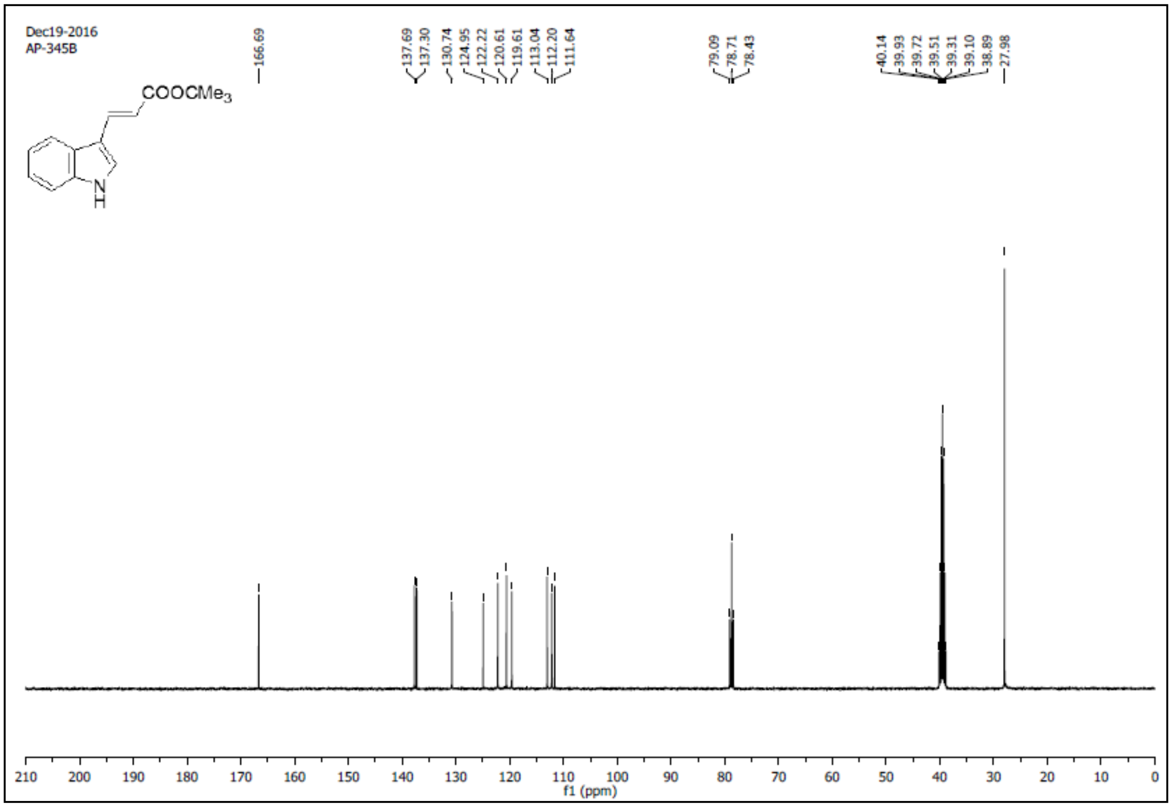

Dec19-2016
AP-345B
166.69
137.69
137.30
130.74
124.95
122.22
120.61
119.61
113.04
112.20
111.64
79.09
78.71
78.43
40.14
39.93
39.72
39.51
39.31
39.10
38.89
27.98
COOCMe3
N
H
f1 (ppm)

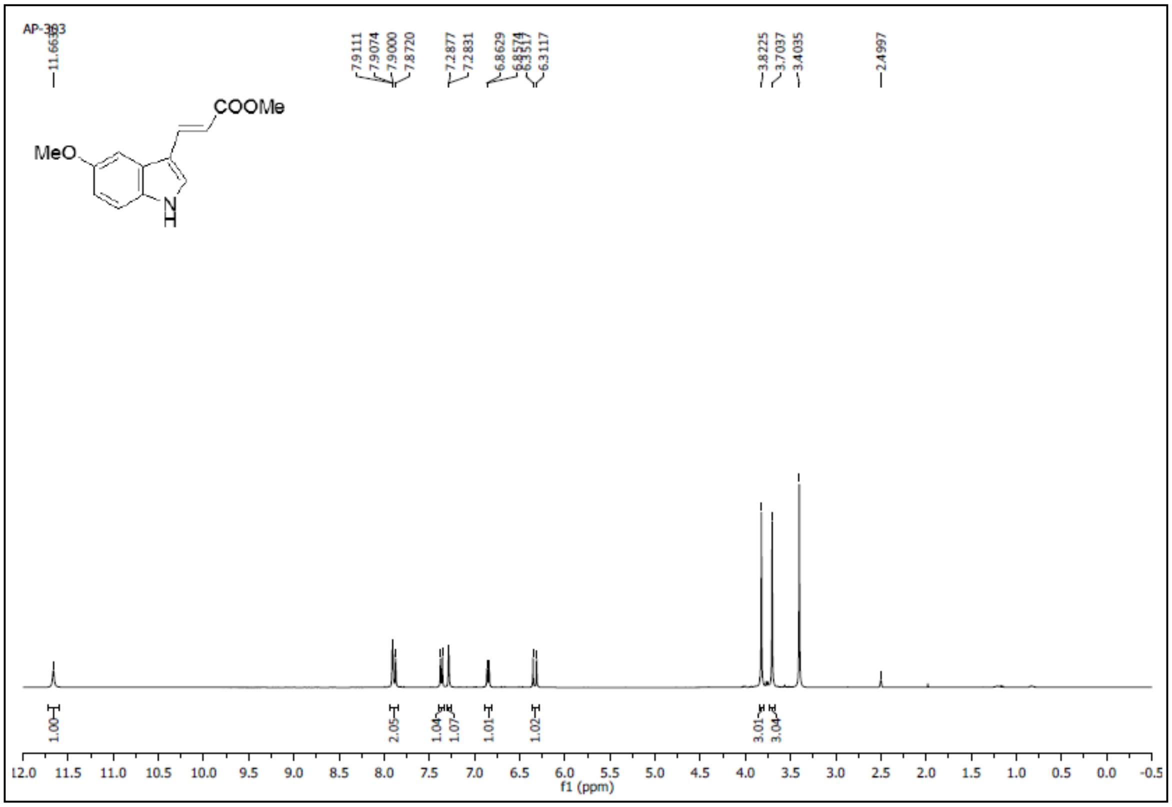
AP-303
11.6639
7.9111
7.9074
7.9000
7.8720
7.2877
7.2831
6.8629
6.8574
6.3517
6.3117
3.8225
3.7037
3.4035
2.4997
COOMe
MeO
N
H
1.00
2.05
1.04
1.07
1.01
1.02
3.01
3.04
f1 (ppm)

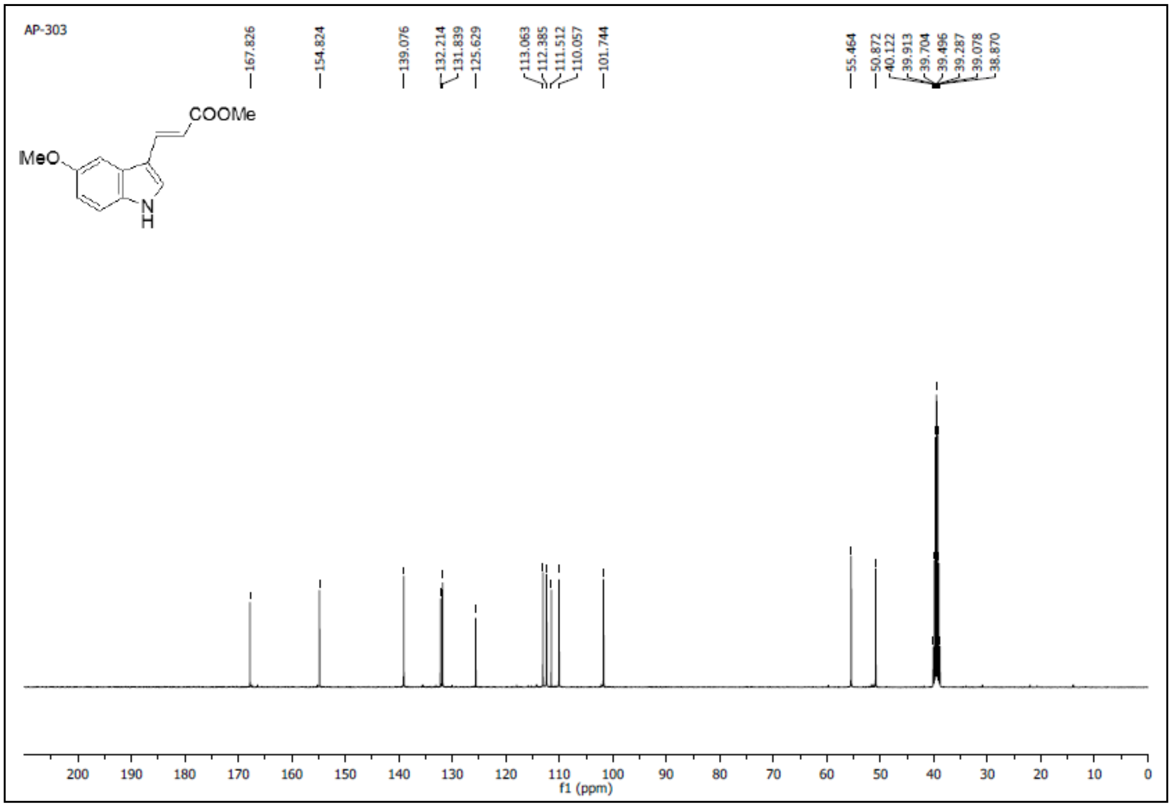
AP-303
167.826
154.824
139.076
132.214
131.839
125.629
113.063
112.385
111.512
110.057
101.744
55.464
50.872
40.122
39.913
39.704
39.496
39.287
39.078
38.870
COOMe
MeO
N
H
f1 (ppm)

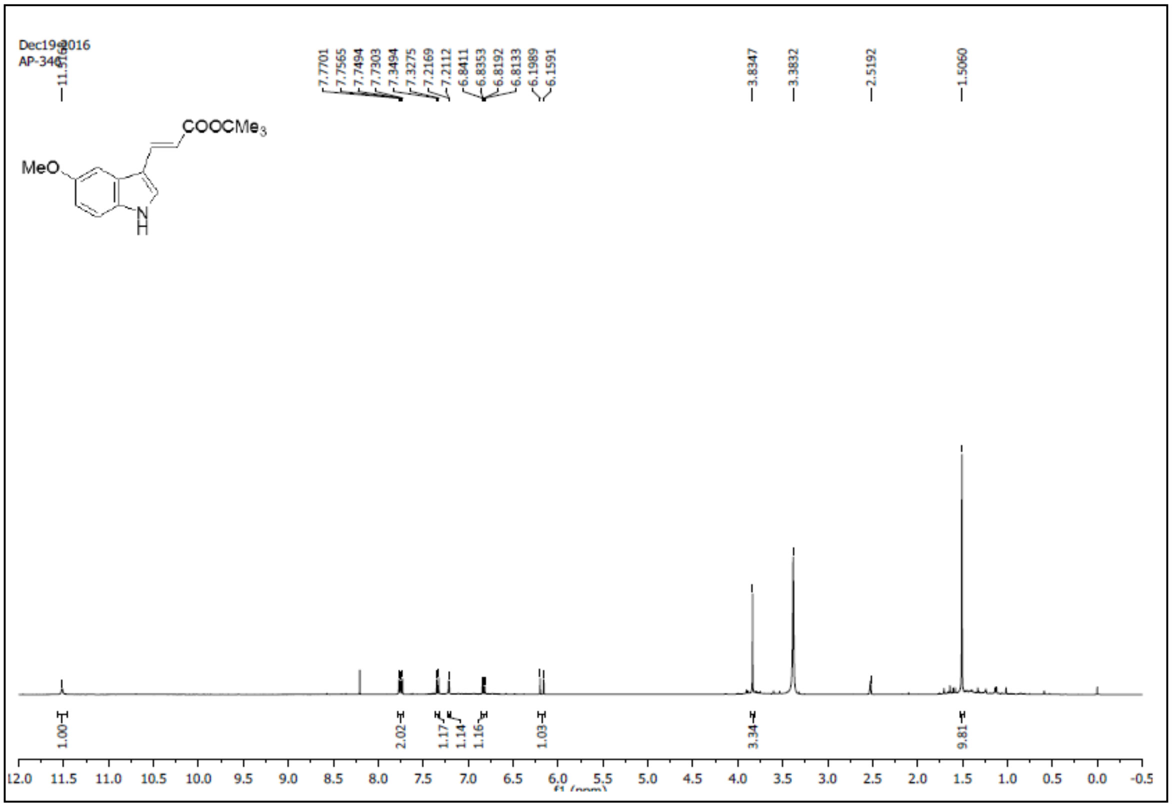
Dec19-2016
AP-346
11.5160
7.7701
7.7565
7.7494
7.7303
7.3494
7.3275
7.2169
7.2112
6.8411
6.8353
6.8192
6.8133
6.1989
6.1591
3.8347
3.3832
2.5192
1.5060
COOCMe3
MeO
N
H
1.00
2.02
1.17
1.14
1.16
1.03
3.34
9.81
f1 (ppm)

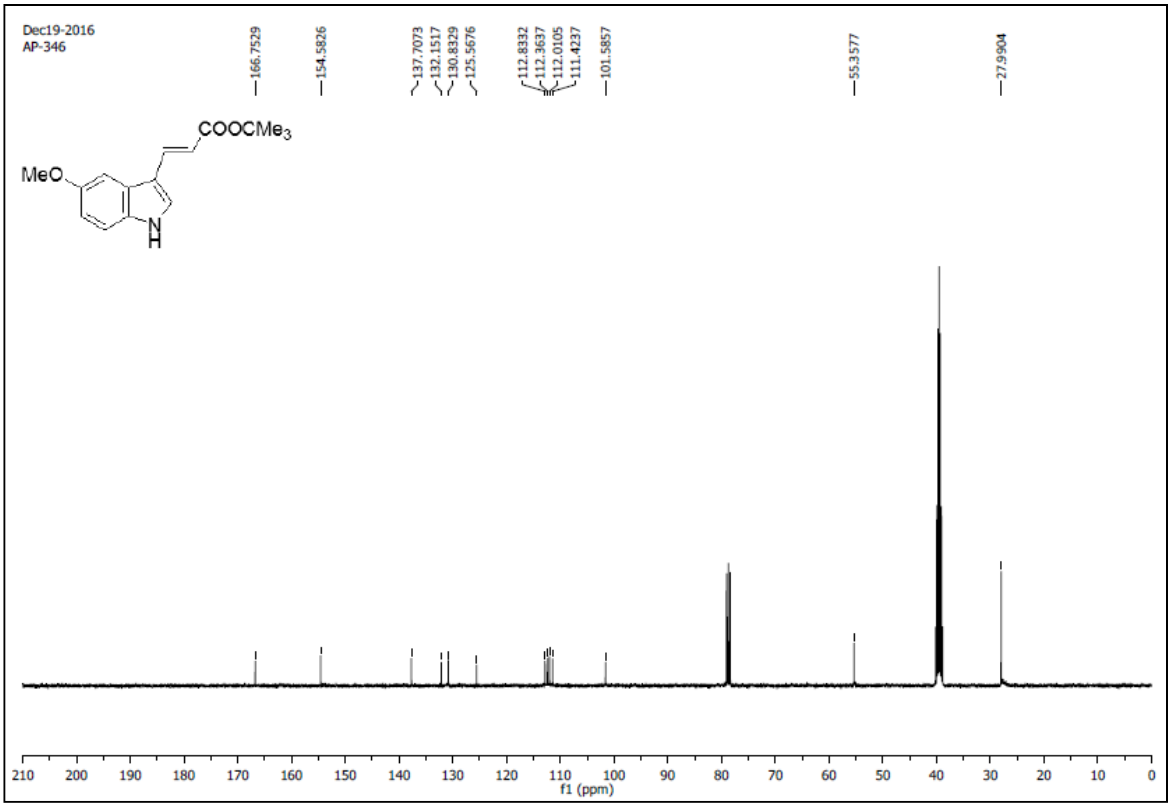
Dec19-2016
AP-346
166.7529
154.5826
137.7073
132.1517
130.8329
125.5676
112.8332
112.3637
112.0105
111.4237
101.5857
55.3577
27.9904
COOCMe3
MeO
N
H
f1 (ppm)

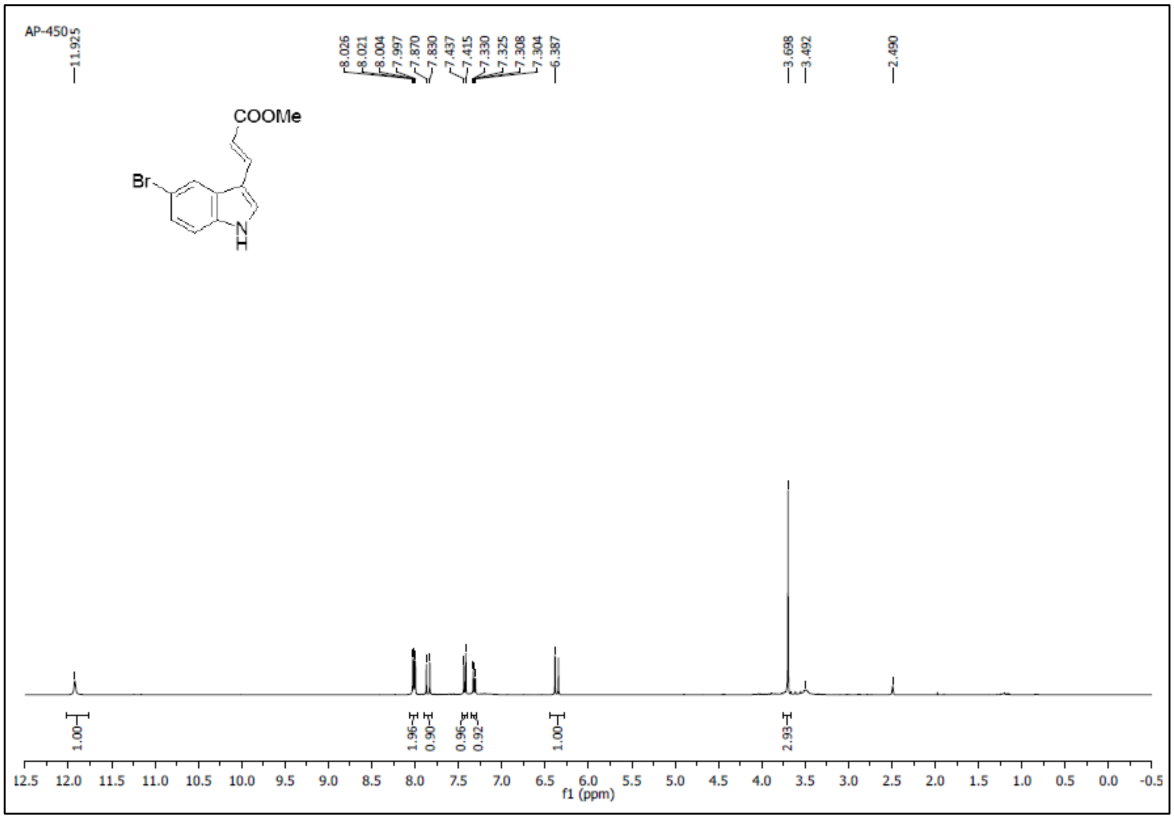
AP-450
11.925
8.026
8.021
8.004
7.997
7.870
7.830
7.437
7.415
7.330
7.325
7.308
7.304
6.387
3.698
3.492
2.490
COOMe
Br
N
H
1.00
1.96
0.90
0.96
0.92
1.00
2.93
12.5 12.0 11.5 11.0 10.5 10.0 9.5 9.0 8.5 8.0 7.5 7.0 6.5 6.0 5.5 5.0 4.5 4.0 3.5 3.0 2.5 2.0 1.5 1.0 0.5 0.0 -0.5
f1 (ppm)

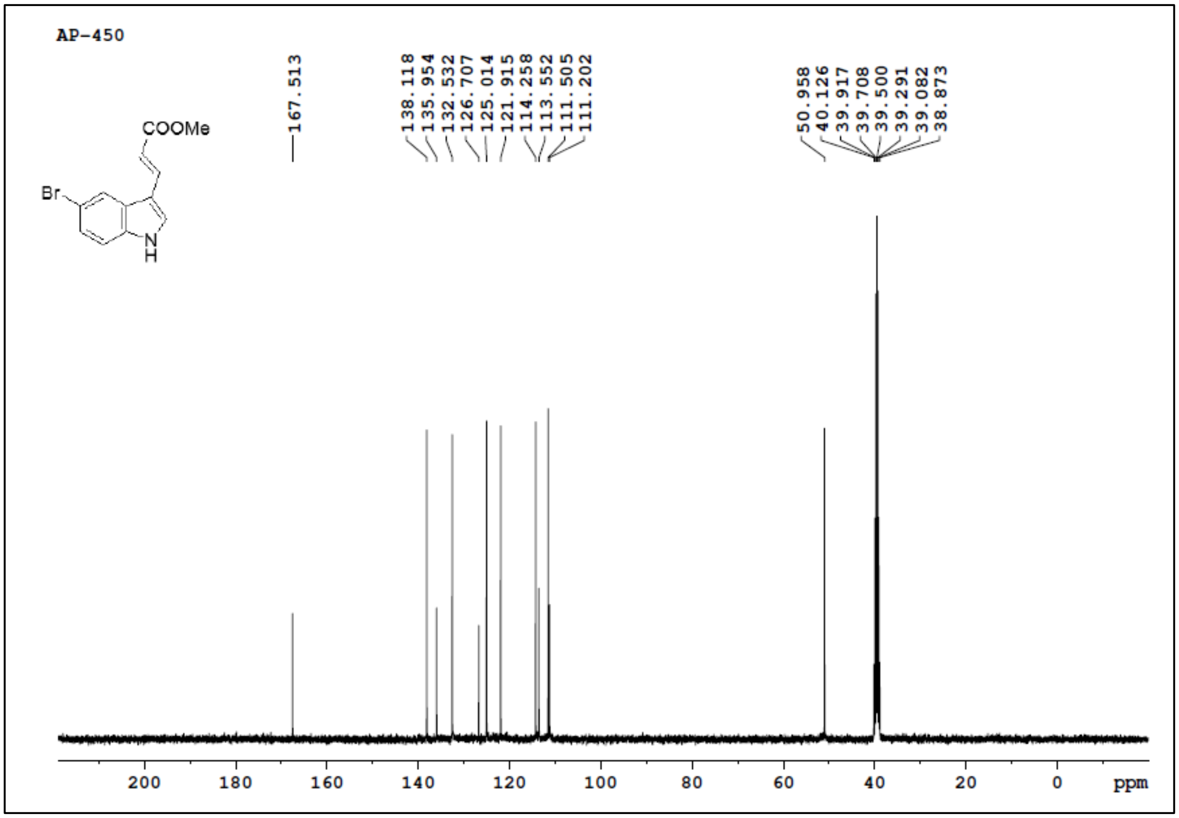
AP-450
167.513
138.118
135.954
132.532
126.707
125.014
121.915
114.258
113.552
111.505
111.202
50.958
40.126
39.917
39.708
39.500
39.291
39.082
38.873
COOMe
Br
N
H
200 180 160 140 120 100 80 60 40 20 0 ppm

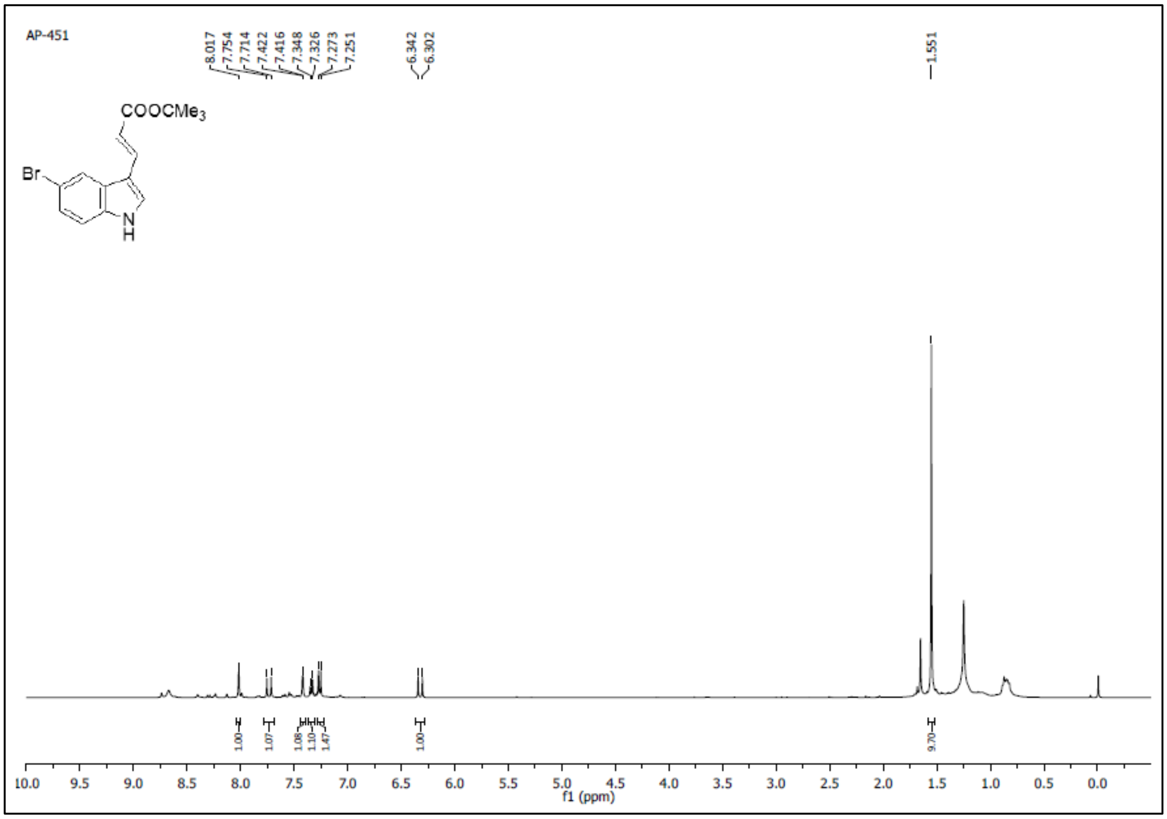
AP-451
COOCMe3
Br
N
H
8.017
7.754
7.714
7.422
7.416
7.348
7.326
7.273
7.251
6.342
6.302
1.551
1.00
1.07
1.08
1.10
1.47
1.00
9.70
10.0 9.5 9.0 8.5 8.0 7.5 7.0 6.5 6.0 5.5 5.0 4.5 4.0 3.5 3.0 2.5 2.0 1.5 1.0 0.5 0.0
f1 (ppm)

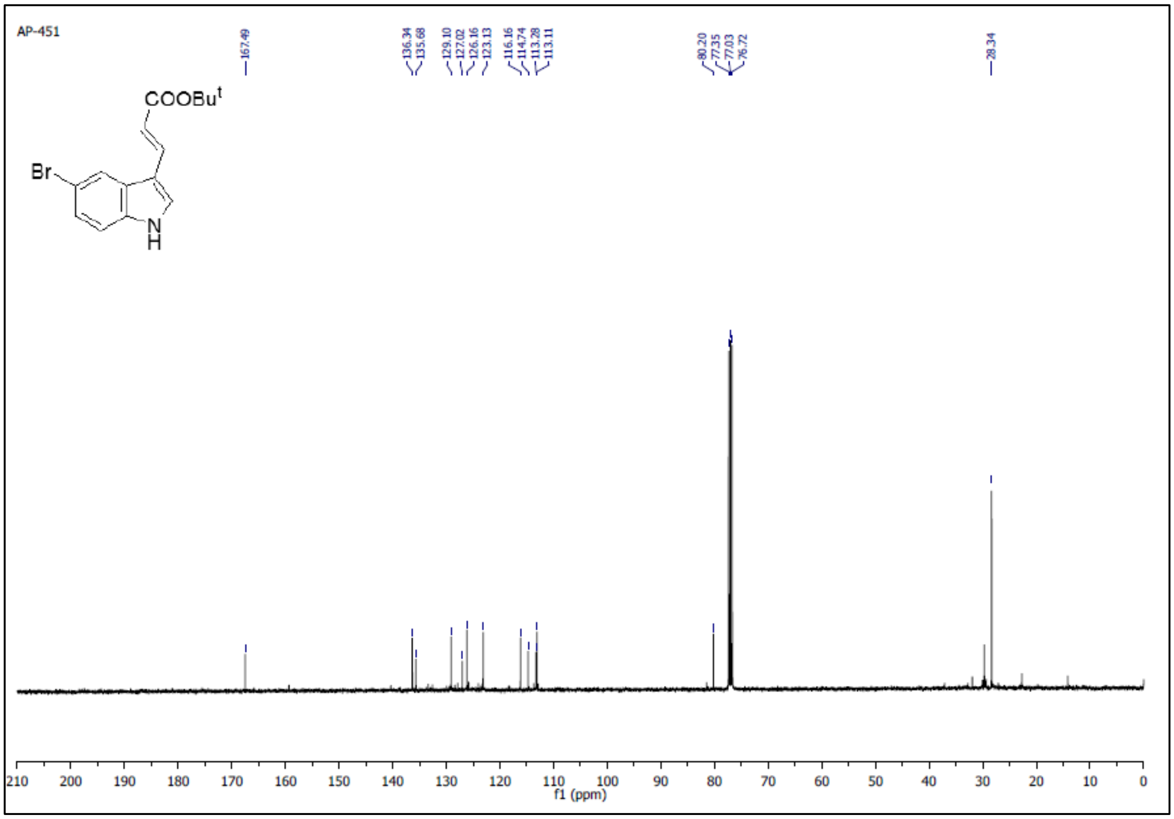
AP-451
COOBut
Br
N
H
167.49
136.34
135.68
129.10
127.02
126.16
123.13
116.16
114.74
113.28
113.11
80.20
77.35
77.03
76.72
28.34
210 200 190 180 170 160 150 140 130 120 110 100 90 80 70 60 50 40 30 20 10 0
f1 (ppm)

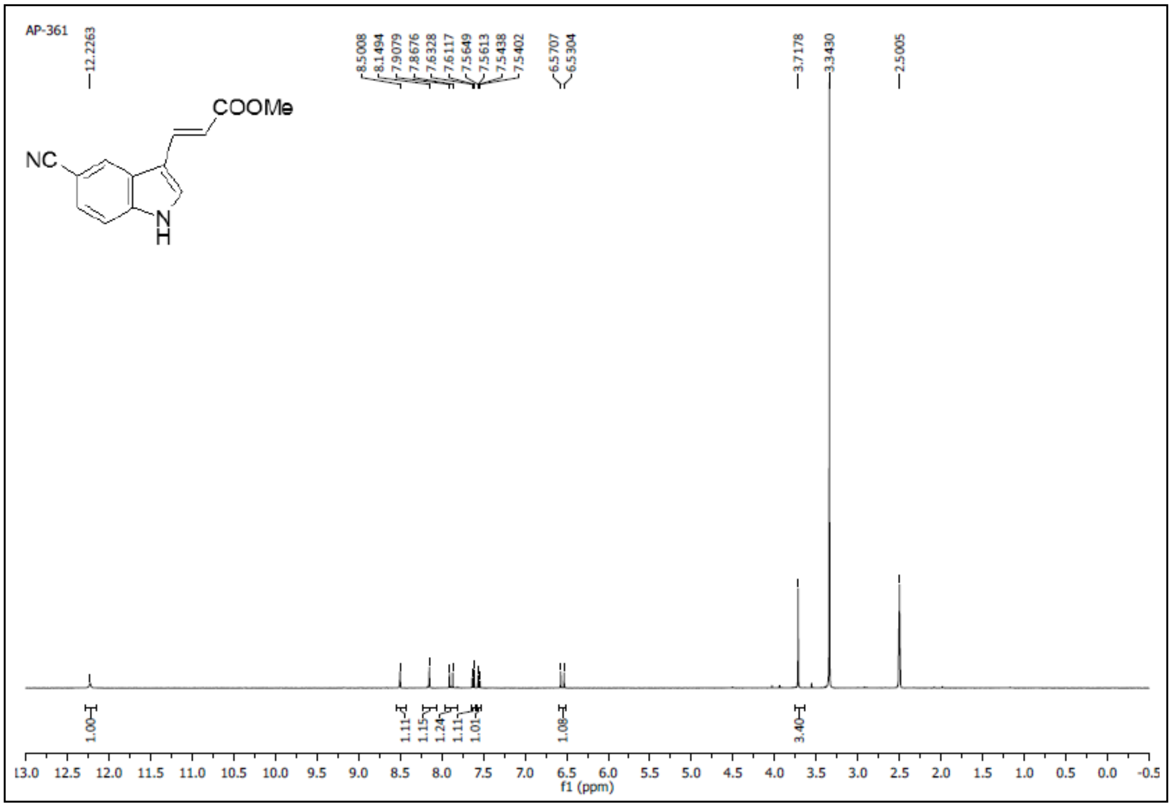
AP-361
12.2263
8.5008
8.1494
7.9079
7.8676
7.6328
7.6117
7.5649
7.5613
7.5438
7.5402
6.5707
6.5304
3.7178
3.3430
2.5005
COOMe
NC
N
H
1.00
1.11
1.15
1.24
1.11
1.01
1.08
3.40
f1 (ppm)

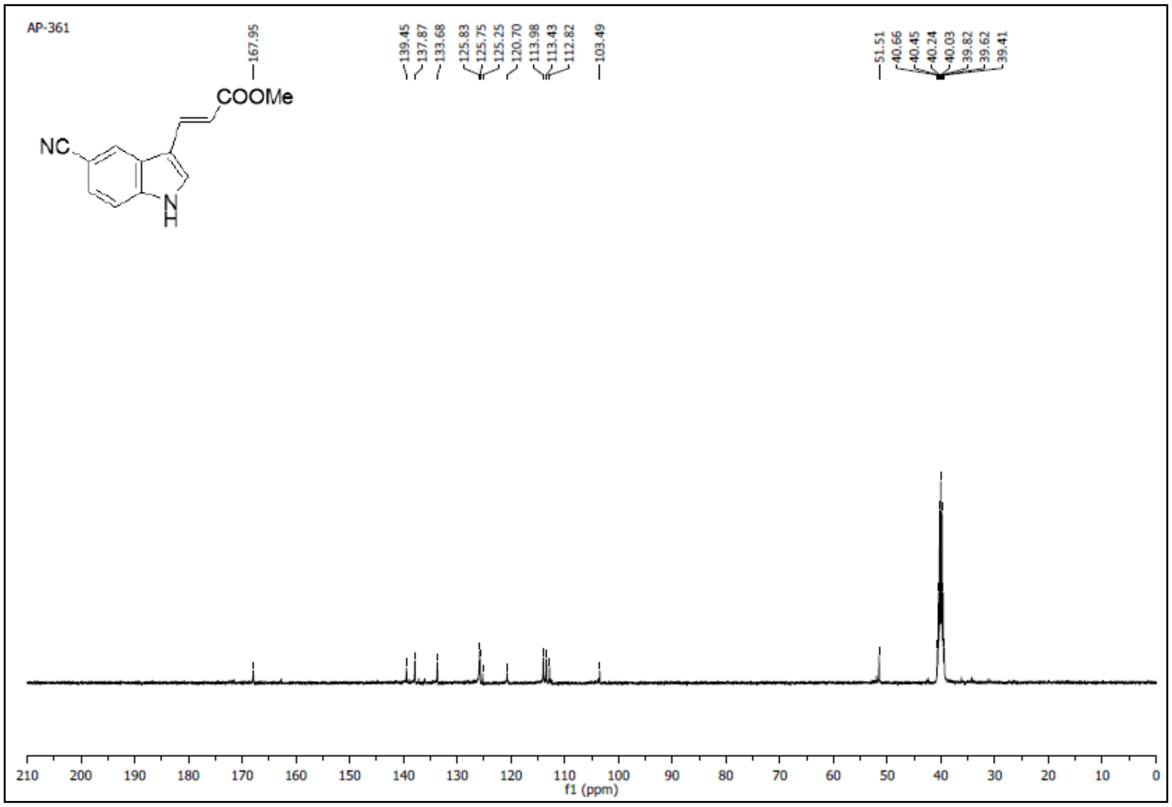
AP-361
167.95
139.45
137.87
133.68
125.83
125.75
125.25
120.70
113.98
113.43
112.82
103.49
51.51
40.66
40.45
40.24
40.03
39.82
39.62
39.41
COOMe
NC
N
H
f1 (ppm)

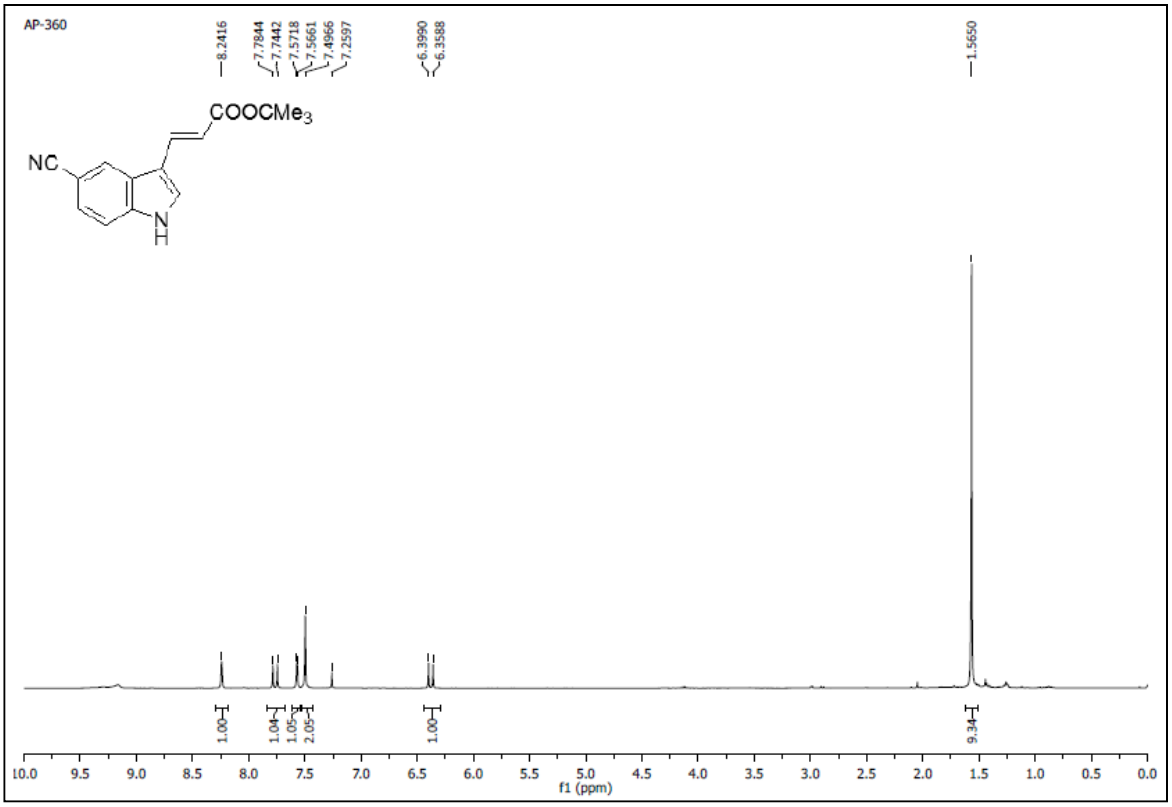
AP-360
8.2416
7.7844
7.7442
7.5718
7.5661
7.4966
7.2597
6.3990
6.3588
1.5650
COOCMe3
NC
N
H
1.00
1.04
1.05
2.05
1.00
9.34
f1 (ppm)

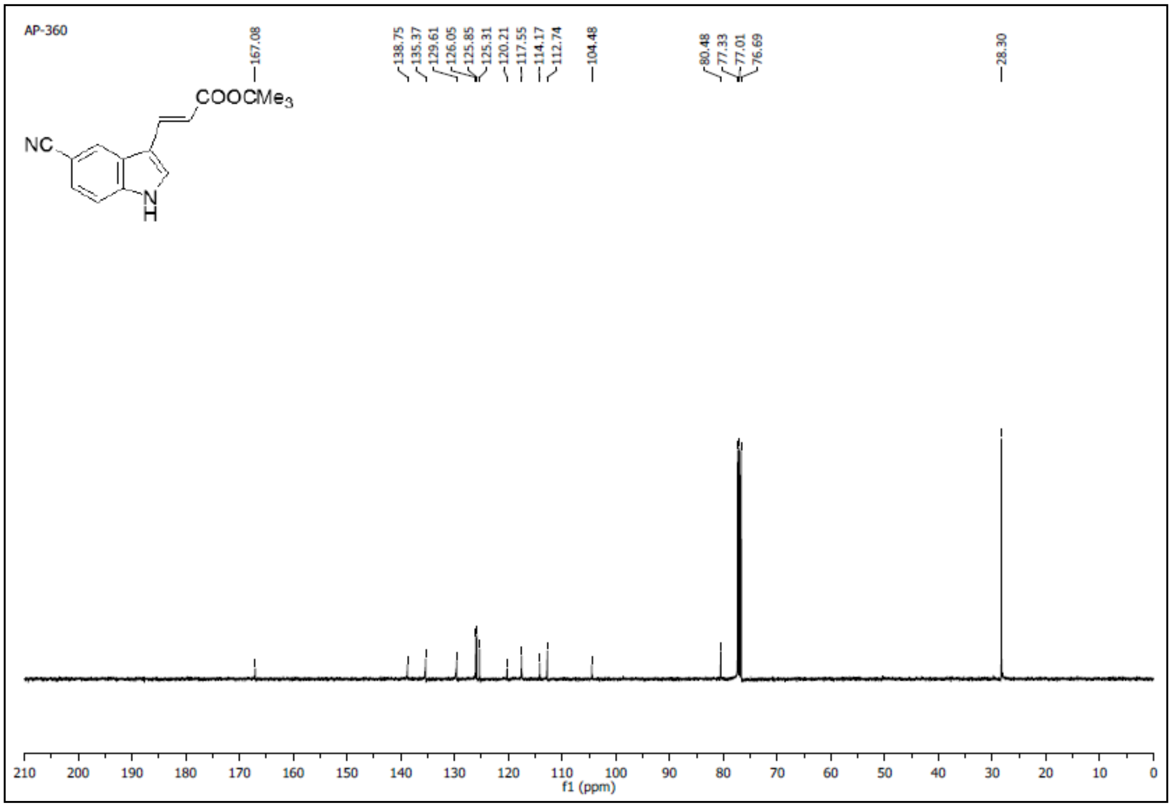
AP-360
167.08
138.75
135.37
129.61
126.05
125.85
125.31
120.21
117.55
114.17
112.74
104.48
80.48
77.33
77.01
76.69
28.30
COOCMe3
NC
N
H
f1 (ppm)

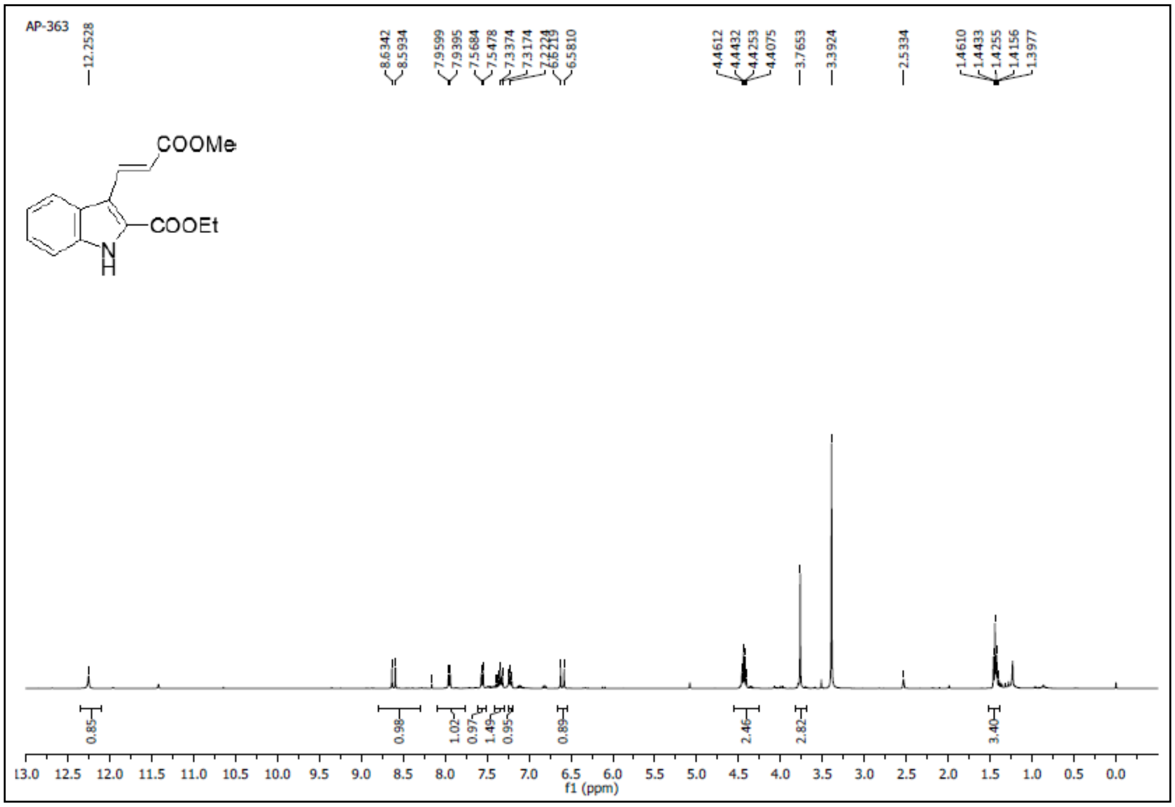
AP-363
COOMe
COOEt
N
H
f1 (ppm)

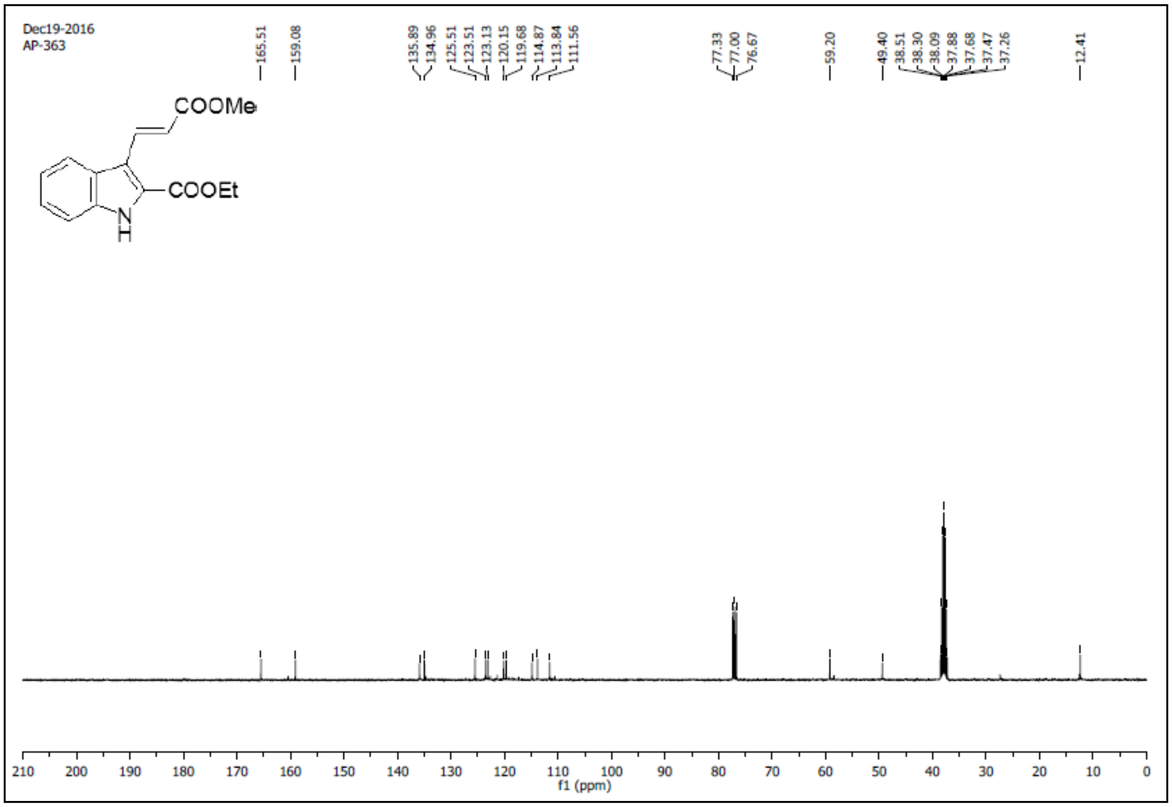
Dec19-2016
AP-363
COOMe
COOEt
N
H
f1 (ppm)

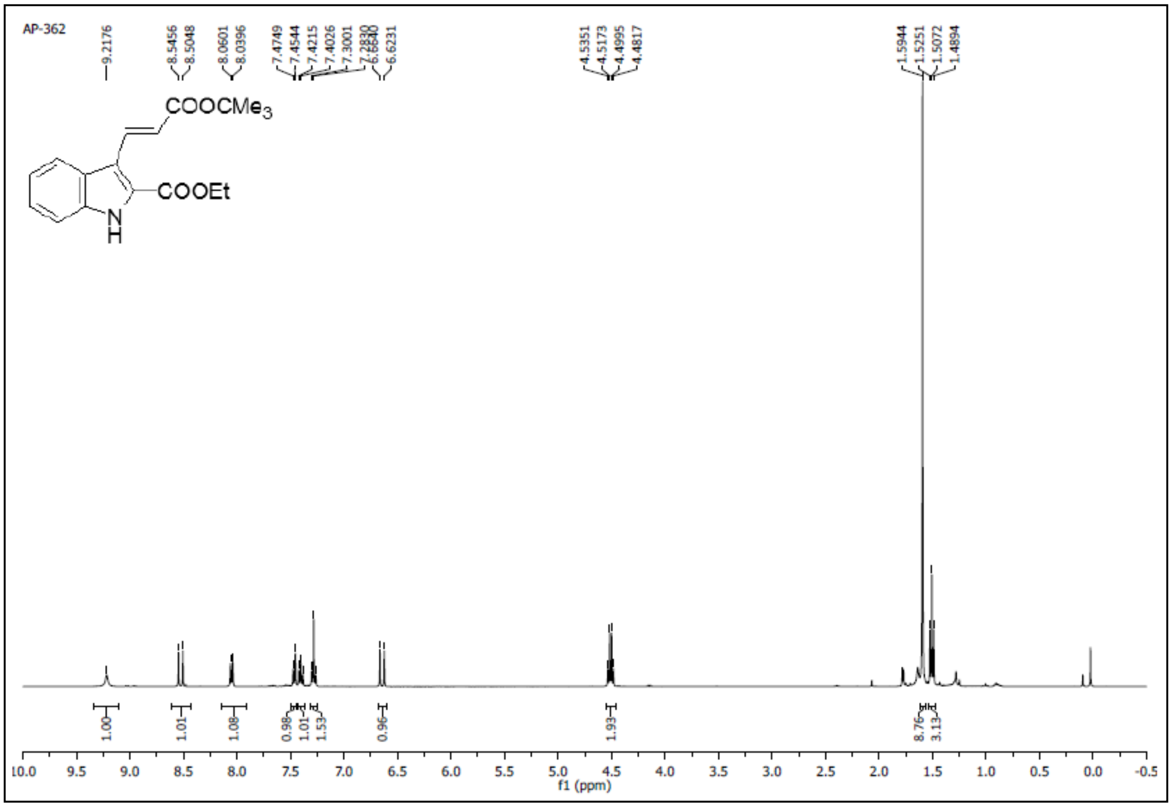
AP-362
9.2176
8.5456
8.5048
8.0601
8.0396
7.4749
7.4544
7.4215
7.4026
7.3001
7.2830
6.6640
6.6231
4.5351
4.5173
4.4995
4.4817
1.5944
1.5251
1.5072
1.4894
COOCMe3
COOEt
N
H
1.00
1.01
1.08
0.98
1.01
1.53
0.96
1.93
8.76
3.13
f1 (ppm)

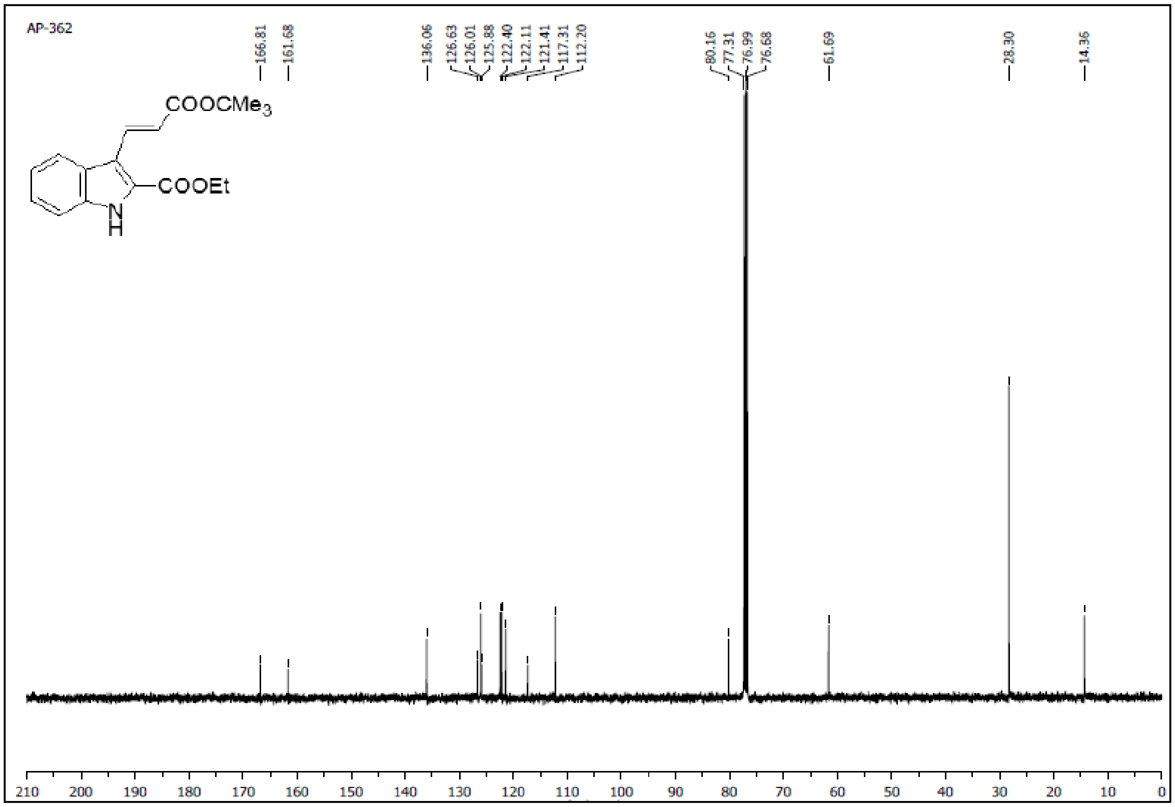
AP-362
166.81
161.68
136.06
126.63
126.01
125.88
122.40
122.11
121.41
117.31
112.20
80.16
77.31
76.99
76.68
61.69
28.30
14.36
COOCMe3
COOEt
N
H

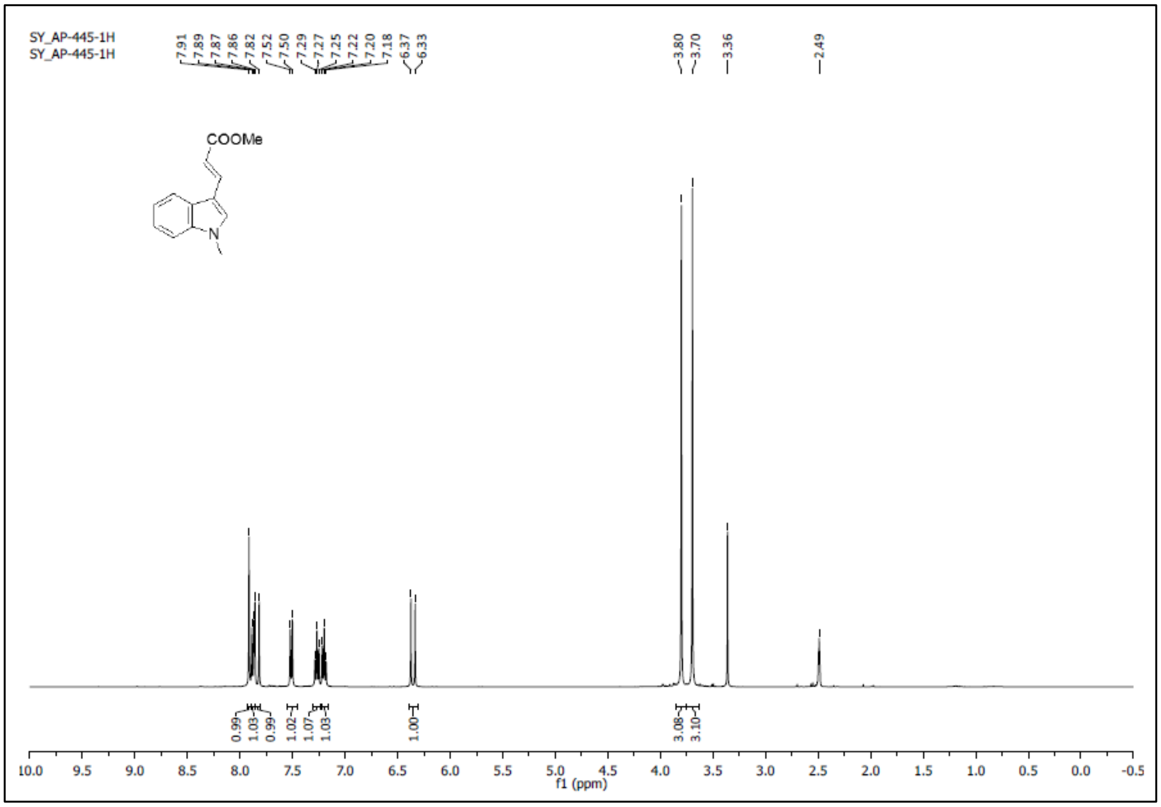
SY_AP-445-1H
SY_AP-445-1H
COOMe
N
f1 (ppm)

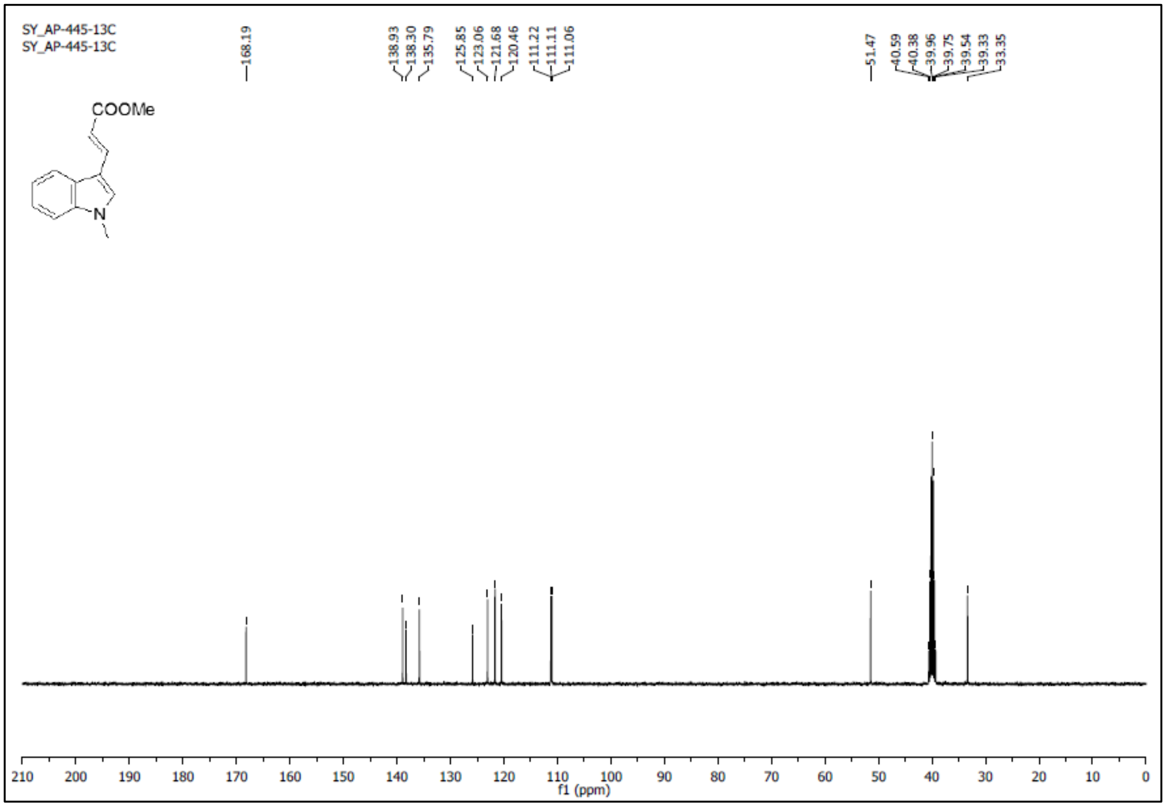
SY_AP-445-13C
SY_AP-445-13C
COOMe
N
f1 (ppm)

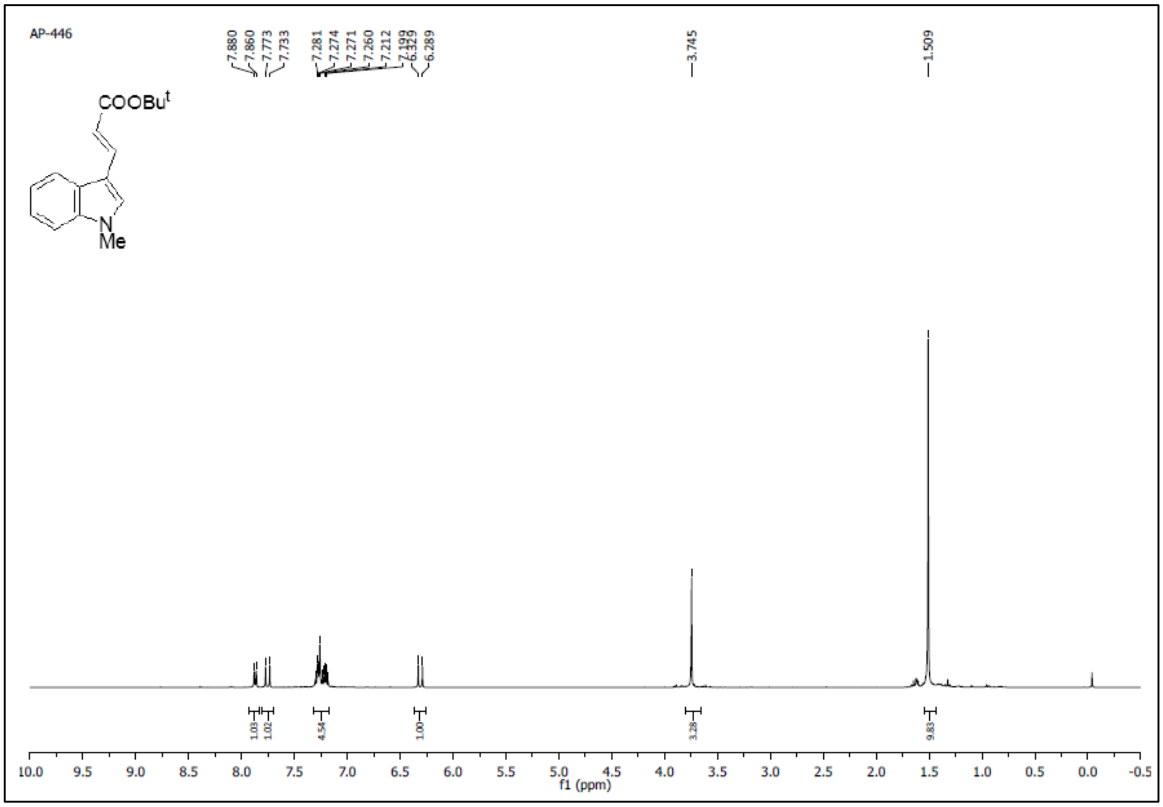
AP-446
COOBu^t
N
Me
7.880
7.860
7.773
7.733
7.281
7.274
7.271
7.260
7.212
7.199
6.329
6.289
3.745
1.509
1.03
1.02
4.54
1.00
3.28
9.83
f1 (ppm)

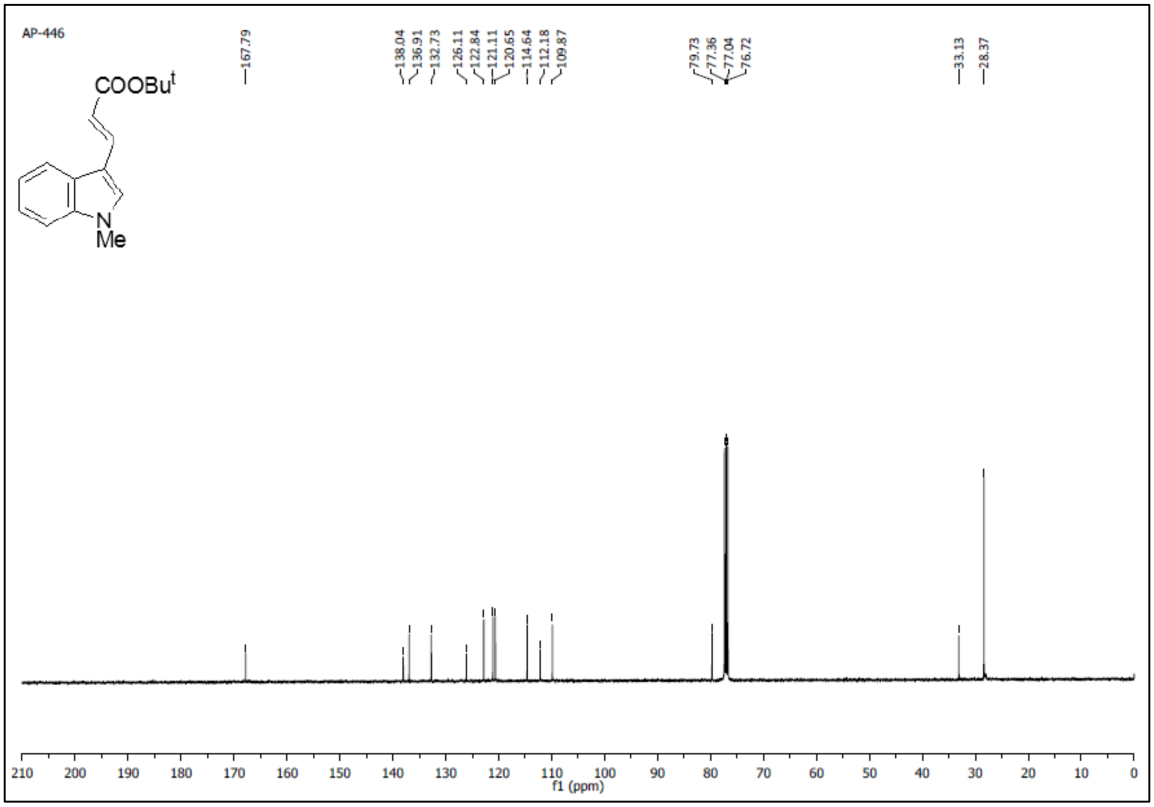
AP-446
COOBu^t
N
Me
167.79
138.04
136.91
132.73
126.11
122.84
121.11
120.65
114.64
112.18
109.87
79.73
77.36
77.04
76.72
33.13
28.37
f1 (ppm)

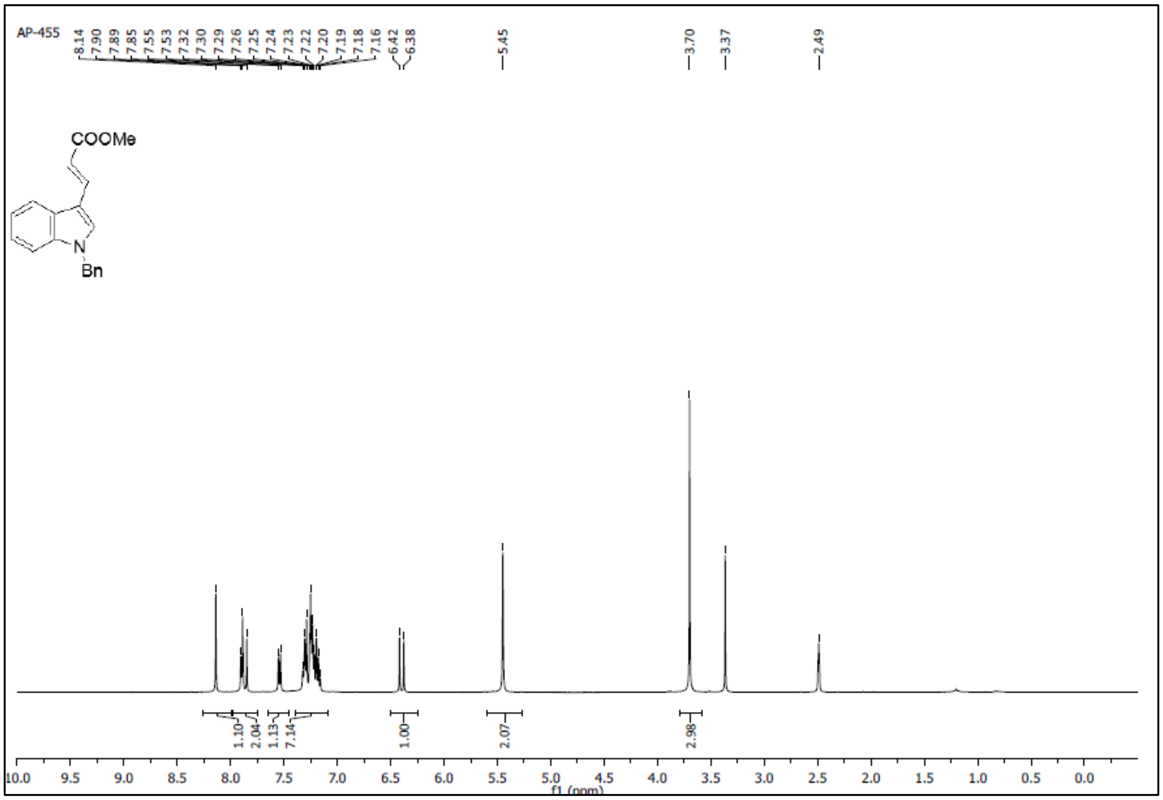
AP-455
8.14
7.90
7.89
7.85
7.55
7.53
7.32
7.30
7.29
7.26
7.25
7.24
7.23
7.22
7.20
7.19
7.18
7.16
6.42
6.38
5.45
3.70
3.37
2.49
COOMe
N
Bn
1.10
2.04
1.13
7.14
1.00
2.07
2.98
10.0
9.5
9.0
8.5
8.0
7.5
7.0
6.5
6.0
5.5
5.0
4.5
4.0
3.5
3.0
2.5
2.0
1.5
1.0
0.5
0.0
f1 (ppm)

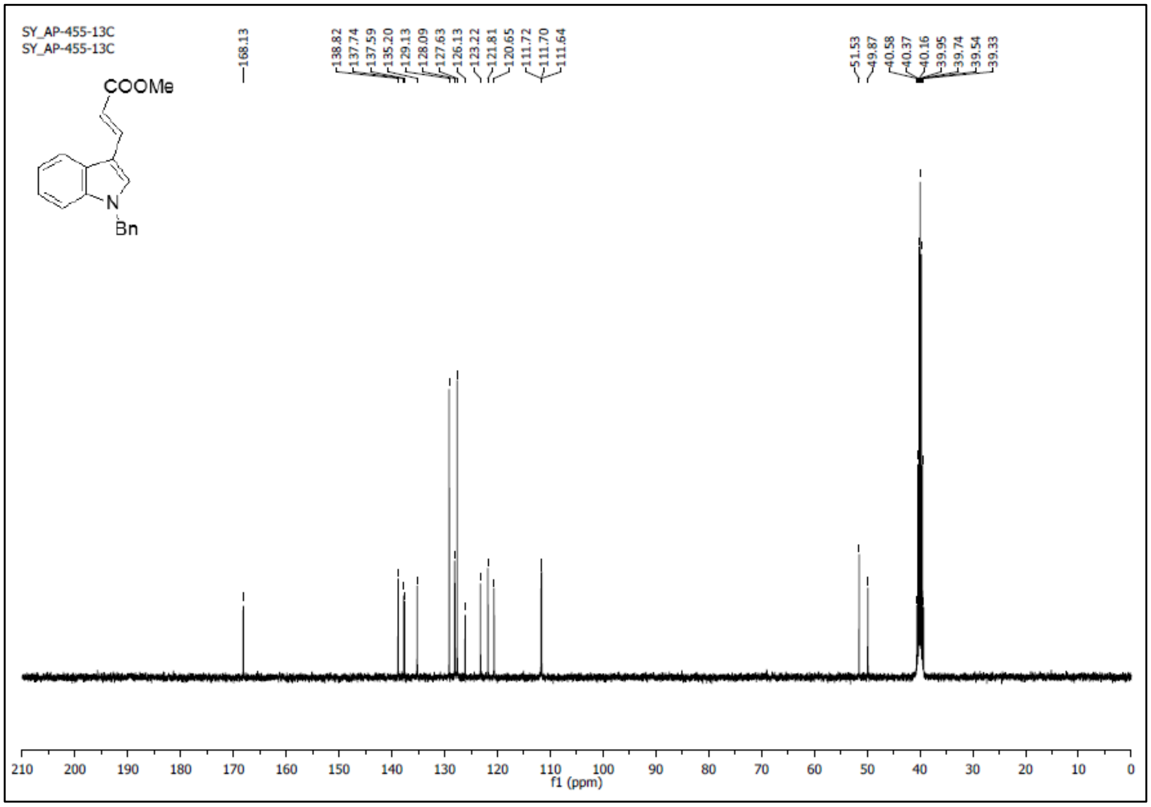
SY_AP-455-13C
SY_AP-455-13C
168.13
138.82
137.74
137.59
135.20
129.13
128.09
127.63
126.13
123.22
121.81
120.65
111.72
111.70
111.64
51.53
49.87
40.58
40.37
40.16
39.95
39.74
39.54
39.33
COOMe
N
Bn
210
200
190
180
170
160
150
140
130
120
110
100
90
80
70
60
50
40
30
20
10
0
f1 (ppm)

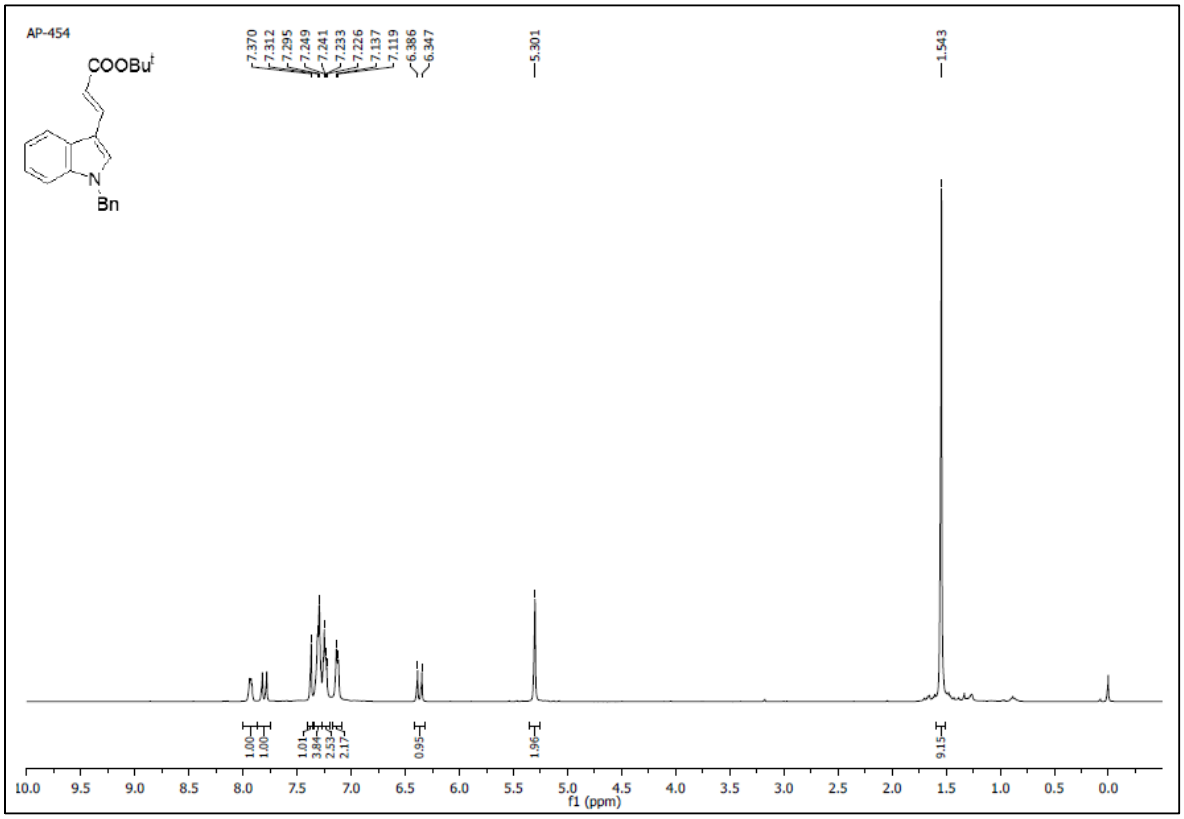
AP-454
COOBu^t
N
Bn
7.370
7.312
7.295
7.249
7.241
7.233
7.226
7.137
7.119
6.386
6.347
5.301
1.543
1.00
1.00
1.01
3.84
2.53
2.17
0.95
1.96
9.15
f1 (ppm)

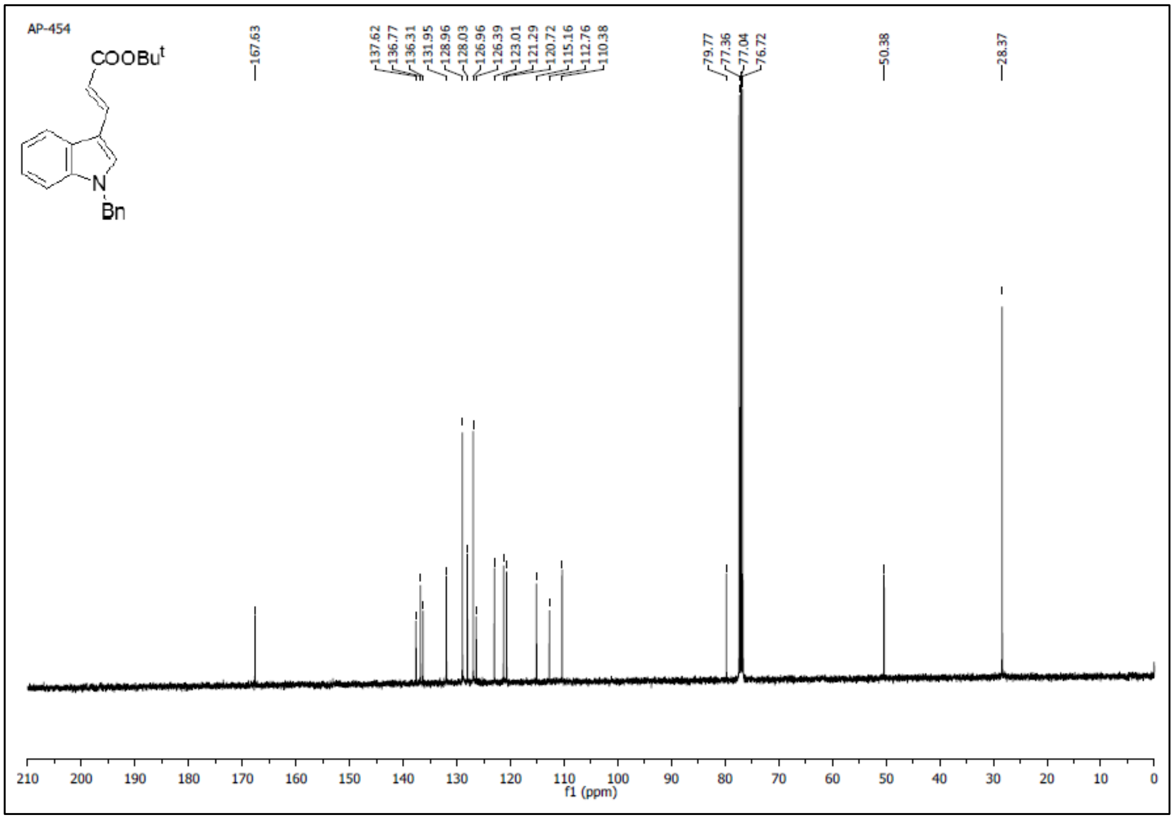
AP-454
COOBu^t
N
Bn
167.63
137.62
136.77
136.31
131.95
128.96
128.03
126.96
126.39
123.01
121.29
120.72
115.16
112.76
110.38
79.77
77.36
77.04
76.72
50.38
28.37
f1 (ppm)

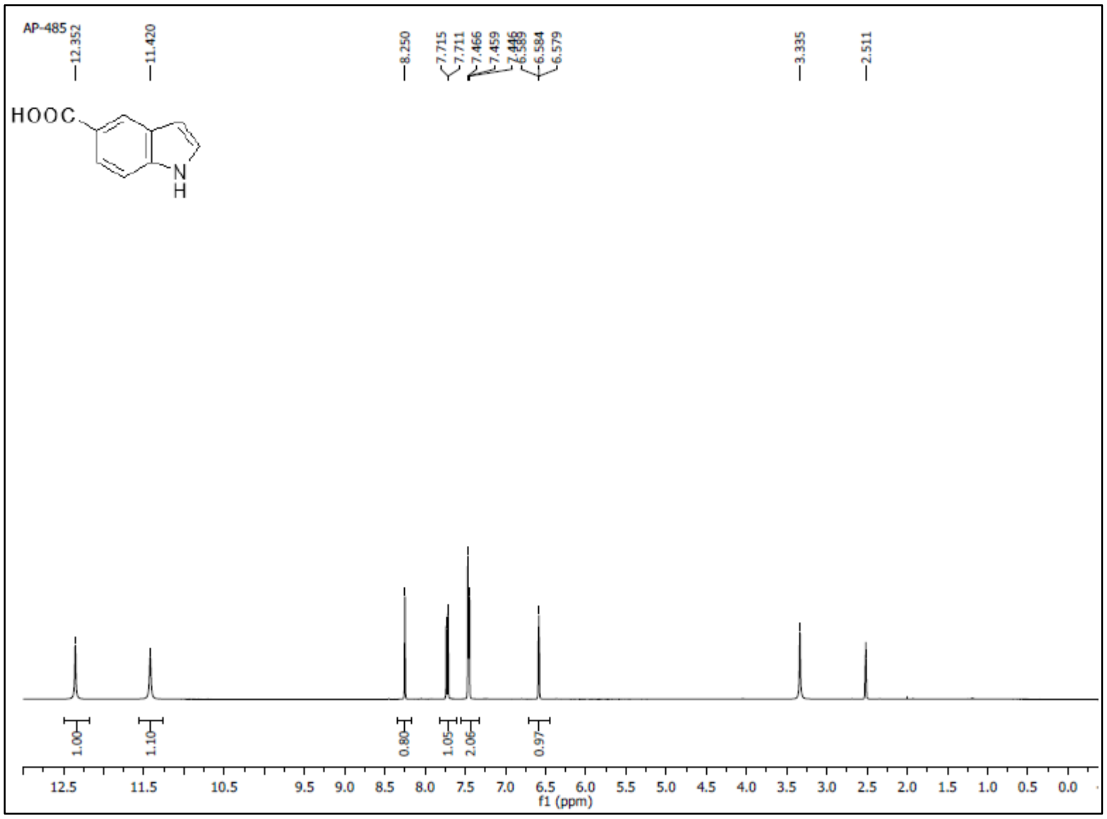
AP-485
12.352
11.420
8.250
7.715
7.711
7.466
7.459
6.584
6.579
3.335
2.511
HOOC
N
H
1.00
1.10
0.80
1.05
2.06
0.97
f1 (ppm)

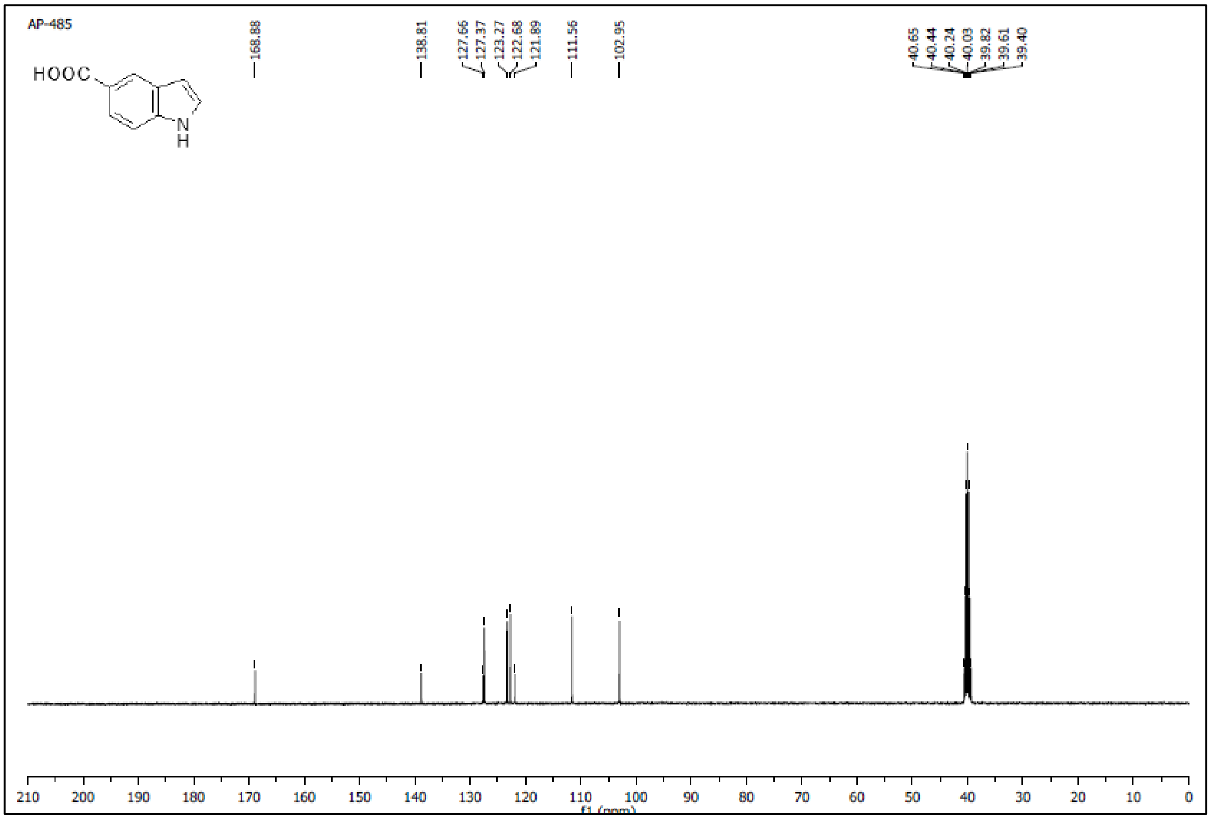
AP-485
168.88
138.81
127.66
127.37
123.27
122.68
121.89
111.56
102.95
40.65
40.44
40.24
40.03
39.82
39.61
39.40
HOOC
N
H

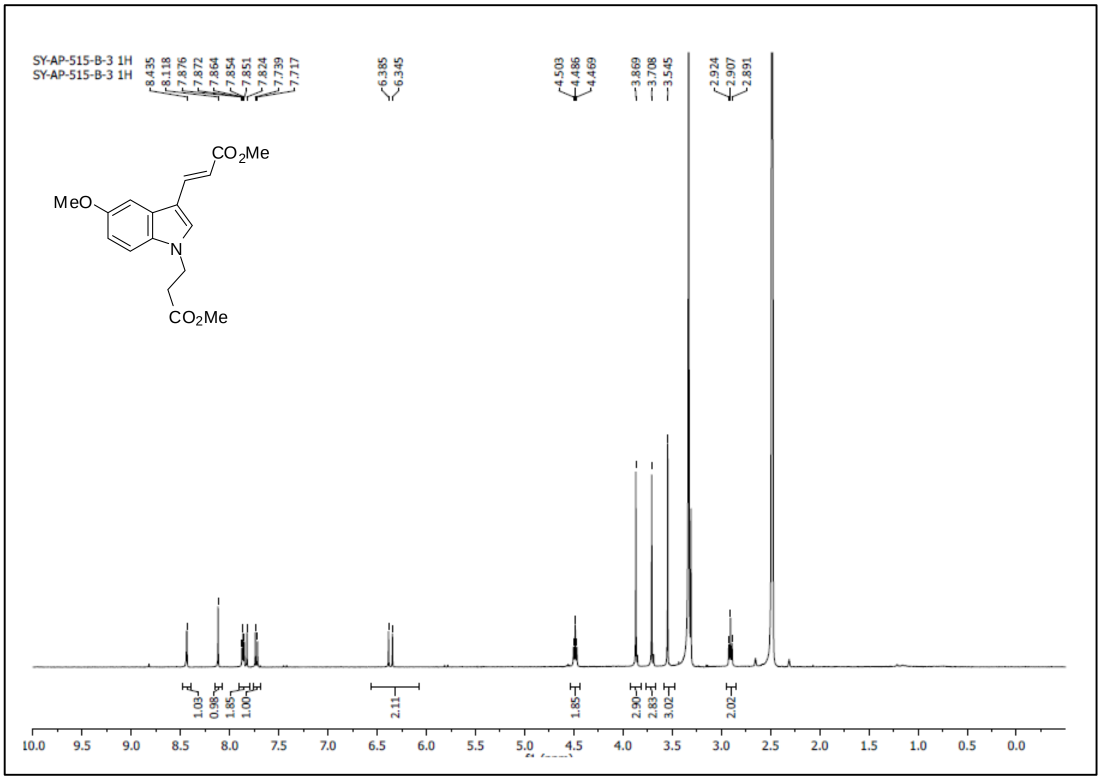
SY-AP-515-B-3 1H
SY-AP-515-B-3 1H
8.435
8.118
7.876
7.872
7.864
7.854
7.851
7.824
7.739
7.717
6.385
6.345
4.503
4.486
4.469
3.869
3.708
3.545
2.924
2.907
2.891
CO2Me
MeO
N
CO2Me
1.03
0.98
1.85
1.00
2.11
1.85
2.90
2.83
3.02
2.02
10.0
9.5
9.0
8.5
8.0
7.5
7.0
6.5
6.0
5.5
5.0
4.5
4.0
3.5
3.0
2.5
2.0
1.5
1.0
0.5
0.0

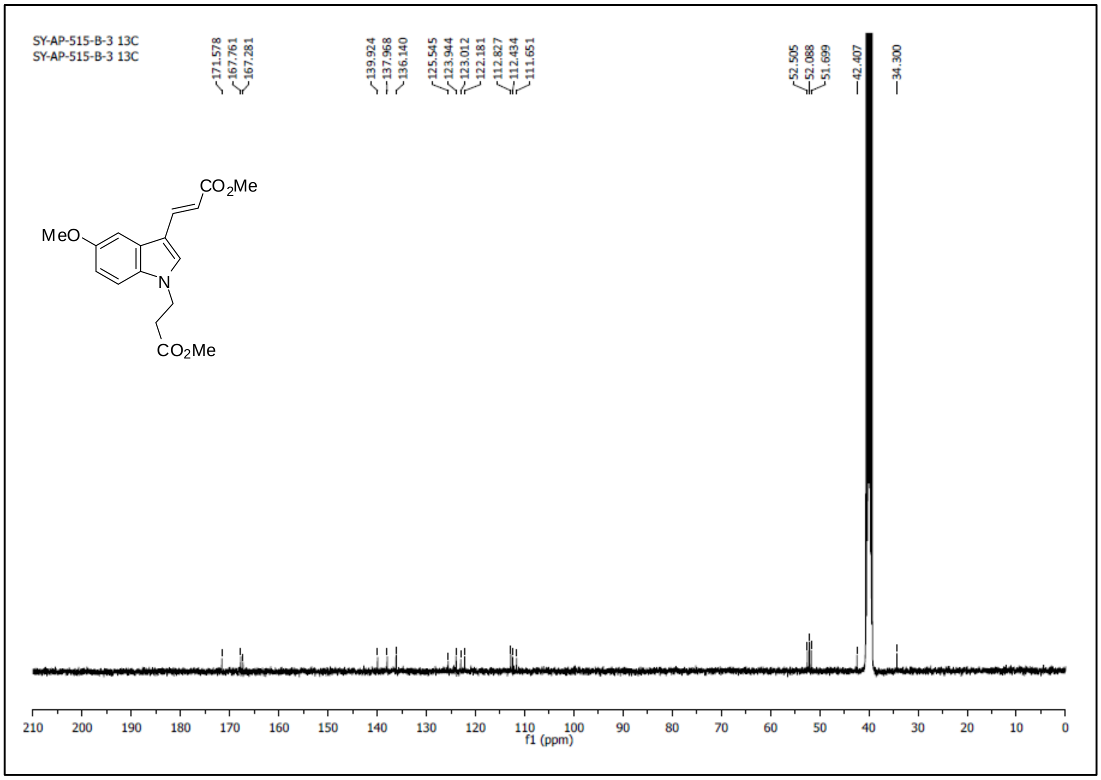
SY-AP-515-B-3 13C
SY-AP-515-B-3 13C
171.578
167.761
167.281
139.924
137.968
136.140
125.545
123.944
123.012
122.181
112.827
112.434
111.651
52.505
52.088
51.699
42.407
34.300
CO2Me
MeO
N
CO2Me
210
200
190
180
170
160
150
140
130
120
110
100
90
80
70
60
50
40
30
20
10
0
f1 (ppm)

References

1 Zhang, S.; Chen, Z.; Qin, S.; Lou, C.; Senan, A. M.; Liao, R. Z.; Yin, G. *Org. Biomol. Chem.* **2016**, *14*, 4146-4157.

2 Huang, Q.; Song, Q.; Cai, J.; Zhang, X.; Lin, S. *Adv. Synth. Catal*. **2013**, *355*, 1512-1516

3 Saunthwal, R. K.; Saini, K. M.; Patel, M.; Verma, A. K. *Tetrahedron* **2017**, 73, 2415-2431.

4 Patent: Heterocyclic derivatives as ROR gamma modulators, WO2016102633.

5 Zhang, Y.; Liu, S.; Yu, W.; Hu, M.; Zhang, G.; Yu, Y. *Tetrahedron* **2013**, *69*, 2070-2074.

6 Chen, H.; Yang, H.; Wang, Z.; Xie, X.; Nan, F. *ACS Med. Chem. Lett.* **2016**, *7*, 335-339.

7 Bhavani, S.; Ashfaq, Md. A.; Rambabu, D.; Rao, M. V. B.; Pal, M. *Arab. J. Chem.* DOI: 10.1016/j.arabjc.2016.02.002

8 Nayal, O. S.; Hong, J.; Yang, Y.; Mo, F. *Org. Chem. Front.* **2019**, *6*, 3673-3677.
